# Therapeutic delivery of microRNA-125a-5p oligonucleotides improves recovery from myocardial ischemia/reperfusion injury in mice and swine

**DOI:** 10.7150/thno.73568

**Published:** 2023-01-01

**Authors:** Ling Gao, Fan Qiu, Hao Cao, Hao Li, Gonghua Dai, Teng Ma, Yanshan Gong, Wei Luo, Dongling Zhu, Zhixuan Qiu, Ping Zhu, Shuguang Chu, Huangtian Yang, Zhongmin Liu

**Affiliations:** 1 Translational Medical Center for Stem Cell Therapy & Institutes for Regenerative Medicine, Shanghai East Hospital, Tongji University School of Medicine, Shanghai 200123, China.; 2 Department of Thoracic Cardiovascular Surgery, The Eighth Affiliated Hospital of Sun Yat-sen University, Shenzhen, Guangdong 518033, China.; 3 Department of Cardiovascular and Thoracic Surgery, Shanghai East Hospital, Tongji University School of Medicine, Shanghai 200120, China.; 4 Department of Radiology, Shanghai East Hospital, Tongji University School of Medicine, Shanghai 200120, China.; 5 Guangdong Cardiovascular Institute, Guangdong Provincial People's Hospital, Guangdong Academy of Medical Sciences, Guangzhou, Guangdong 510100, China.; 6 Research Institute of Heart Failure, Shanghai East Hospital, Tongji University School of Medicine, Shanghai 200120, China.; 7 CAS Key Laboratory of Tissue Microenvironment and Tumor, Laboratory of Molecular Cardiology, Shanghai Institute of Nutrition and Health, University of Chinese Academy of Sciences (CAS), CAS, Shanghai 200031, China.; 8 Shanghai Institute of Stem Cell Research and Clinical translation, Shanghai East Hospital, Tongji University, Shanghai 200120, China.

**Keywords:** myocardial ischemia/reperfusion, macrophage polarization, fibrosis, angiogenesis, swine

## Abstract

**Rationale:** Clinical application of mesenchymal stem cells (MSCs) and MSC-derived exosomes (MSC-Exos) to alleviate myocardial ischemia/reperfusion (I/R) injury is compromised by the low cell engraftment rate and uncontrolled exosomal content. As one of their active ingredients, single-component microRNA therapy may have more inherent advantages. We sought to find an ideal microRNA candidate and determine whether it could reproduce the cardioprotective effects of MSCs and MSC-Exos.

**Methods:** Cardiac function and myocardial remodeling in MSC, MSC-Exo, or microRNA oligonucleotide-treated mouse hearts were investigated after I/R injury. The effects of microRNA oligonucleotides on cardiac cells (macrophages, cardiomyocytes, fibroblasts, and endothelial cells) and their downstream mechanisms were confirmed. Large animals were also employed to investigate the safety of microRNA therapy.

**Results:** The results showed that microRNA-125a-5p (miR-125a-5p) is enriched in MSC-Exos, and intramyocardial delivery of their modified oligonucleotides (agomir) in mouse I/R myocardium, as well as MSCs or MSC-Exos, exerted obvious cardioprotection by increasing cardiac function and limiting adverse remodeling. In addition, miR-125a-5p agomir treatment increased M2 macrophage polarization, promoted angiogenesis, and attenuated fibroblast proliferation and activation, which subsequently contributed to the improvements in cardiomyocyte apoptosis and inflammation. Mechanistically, *Klf13*, *Tgfbr1*, and *Daam1* are considered the targets of miR-125a-5p for regulating the function of macrophages, fibroblasts, and endothelial cells, respectively. Similar results were observed following miR-125a-5p agomir treatment in a porcine model, with no increase in the risk of arrhythmia or hepatic, renal, or cardiac toxicity.

**Conclusions:** This targeted microRNA delivery presents an effective and safe strategy as a stem cell and exosomal therapy in I/R cardiac repair.

## Introduction

Although multiple pathophysiological properties congregate to remodel the heart after myocardial infarction (MI), the fundamental determinants of this process (and its progression to clinical heart failure) are the extent of the initial infarct size and the sufficiency of the reparative process after MI. In clinical practice, limiting infarct size is routinely achieved by timely coronary reperfusion. However, this ischemia/reperfusion (I/R) process also triggers a robust inflammatory cascade in the heart and contributes to the final infarct size 1. Mesenchymal stem cell (MSC) therapy has well-established angiogenic and anti-inflammatory roles in the I/R heart 2, and most of the conclusive benefits are primarily attributed to the secreted exosomes that contain biologically active microRNAs and proteins 3, 4. As the main active ingredient in MSC-Exos, microRNA synthetic oligonucleotides (microRNA mimic or agomir) have many innate advantages, including a single component, absence of immunogenicity, and convenience for production and transportation 5. Nevertheless, whether microRNA synthetic oligonucleotides can reproduce the effect of MSC or MSC-Exo treatment in the I/R myocardium is not yet understood.

It is well established that macrophages show phasic functional heterogeneity and play an important role in the regulation of the intense sterile inflammation of myocardial I/R 6. After reperfusion, M1 macrophage infiltration, which serves to digest and clear damaged cells and cell debris, is increased, followed by a reparative phase with a predominance of M2 macrophages; this phase shows an elevated expression of anti-inflammatory, profibrotic, and growth factors, which are essential for wound healing and scar formation 7, 8. Persistent chronic inflammation and macrophage infiltration have also been observed in the myocardium weeks to months after I/R, and have been associated with cardiac remodeling and heart failure 9. Although early inflammatory activation is a necessary event for the transition to the later reparative program, proper and timely repression and resolution of acute inflammation are determinants of the quality of cardiac repair 9. Considering this, an appropriate balance switching between M1 and M2 macrophages, achieved by earlier and more prevalent M2 macrophage infiltration, may be a therapeutic strategy for myocardial I/R injury. Previous studies have reported that the administration of MSCs or MSC-Exos inhibits myocardial I/R injury by promoting the polarization of macrophages toward the anti-inflammatory M2 phenotype 10, 11. Interestingly, our microRNA sequencing results showed that microRNA-125a-5p (miR-125a-5p) is one of the most highly expressed microRNAs in MSC-Exos and has also been reported to be enriched in M2 macrophages 12, suggesting that miR-125a-5p may be involved in the regulation of MSC-Exo-mediated I/R cardiac macrophage polarization. Additionally, miR-125a-5p has also been suggested to have a protective role in heart failure, as its level is downregulated in the serum of patients with advanced heart failure and negatively correlated with the levels of heart failure biomarkers 13. However, the roles and association of miR-125a-5p on I/R myocardial protection and macrophage polarization remain to be identified. Additionally, the effects of miR-125a-5p on other cardiac cells (e.g., cardiomyocytes, fibroblasts, and endothelial cells) in the I/R heart need further investigation.

The swine has cardiac and coronary anatomy close to those of humans, and the porcine model of regional myocardial I/R closely resembles the human situation, which is of pivotal translational value for cardiovascular research 14. The risk of arrhythmia is critical to the safety of myocardial I/R therapy, and it has been reported that the pro-proliferative microRNA-199a (miR-199a) caused severe arrhythmic death in swine when transfected into porcine I/R myocardium through adeno-associated virus serotype 6 vector 15. Thus, investigating the safety of microRNA therapy, especially in terms of arrhythmia, in large animals is important for clinical translation. Additionally, considering the rapid degradation of microRNA mimics after *in vivo* injection 16, we used a synthetic microRNA agomir (2'OMe + 5'chol modified) to imitate the function of endogenous microRNA. This synthetic microRNA agomir possesses good stability and enhanced transfection efficiency in cells and tissues to enrich target cells and represents an ideal tool for microRNA delivery in animals 17-19.

The goals of this study were as follows: (i) to investigate whether the protective role of MSCs and MSC-Exos on myocardial I/R injury can be reproduced by miR-125a-5p agomir in mice; (ii) to test whether miR-125a-5p protects I/R hearts by modulating macrophage polarization or interacting with other cells, as well as to elucidate their downstream mechanisms; and (iii) to confirm the efficacy and safety of miR-125a-5p agomir treatment in a translational porcine model with cardiac I/R.

## Materials and Methods

The experimental details are provided in the online Supplemental Information.

## Results

### Characterization of MSCs and MSC-Exos

Mouse bone marrow-derived MSCs were isolated and identified by flow cytometry (FCM) analysis as CD73, CD90, and CD105 positive and CD14, CD34, CD45, and human leukocyte antigen class II-DR (HLA-DR) negative 20 (**Figure S1A**). The osteogenic, chondrogenic, and adipogenic abilities of the isolated-MSCs were also determined to verify the cell multipotency (**Figure S1B-S1D**). Next, the exosomes were collected and concentrated from the culture medium of MSCs through differential ultracentrifugation to assess the particle size and concentration (by nanoparticle tracking analysis [NTA]), as well as the morphology and exosome marker expression (**Figure S2A-S2D**). The mean particle diameter for MSC-Exos was ≈ 91 nm, and they expressed recognized exosome markers, including CD63, CD9, tumor susceptibility gene 101 protein (TSG101), and ALG-2-interacting protein X (Alix).

### Delivery of agomir of MSC-Exo-enriched miR-125a-5p following myocardial I/R injury improves mouse myocardial contractile function and limits cardiac remodeling

**Figure 1:**
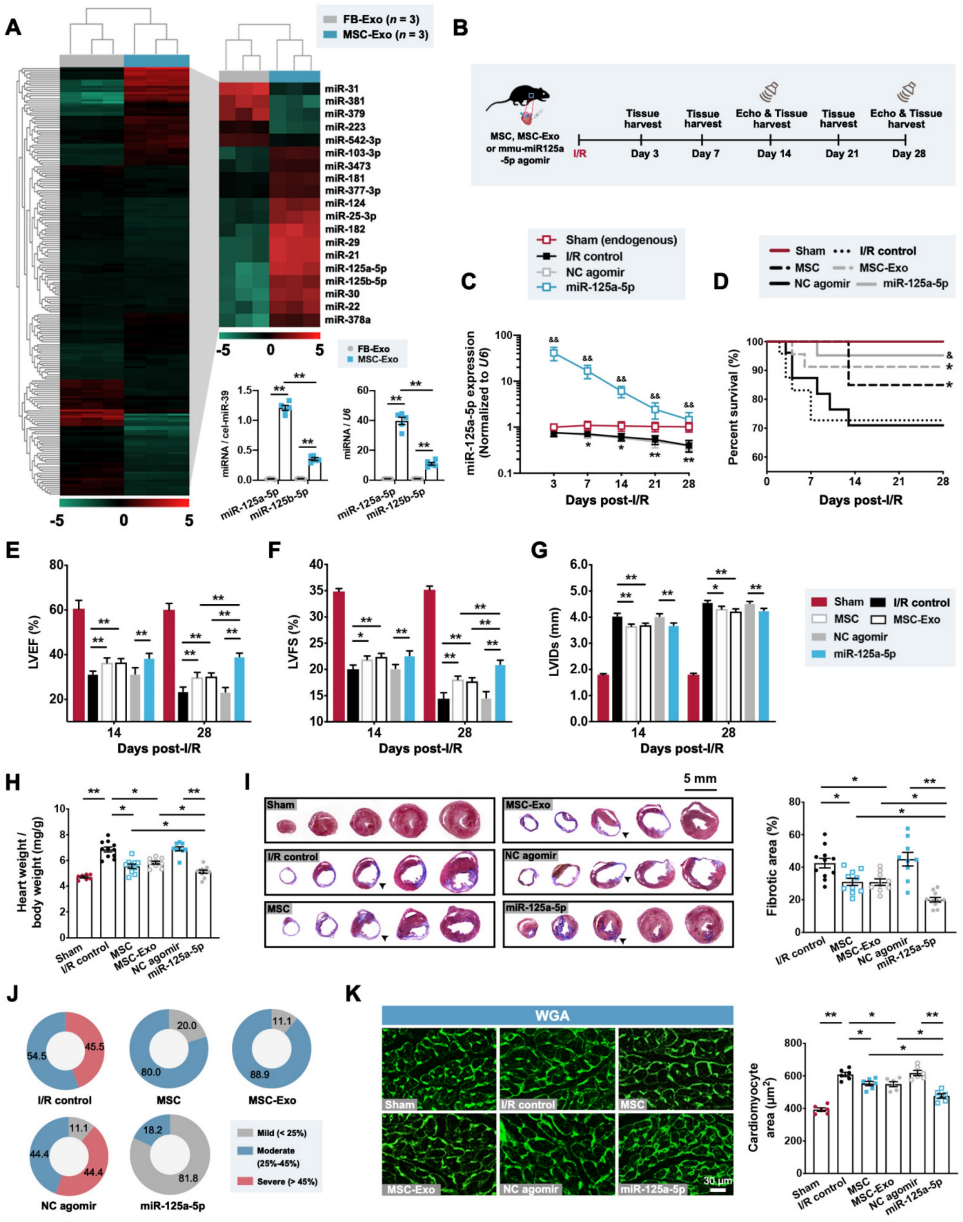
** Administration of the agomir of MSC-derived exosomal microRNA-125a-5p (miR-125a-5p) improves cardiac function and limits myocardial remodeling in a mouse myocardial ischemia/reperfusion (I/R) model.** (**A**) MicroRNA sequencing heat map comparing the microRNA abundance of mouse cardiac fibroblast-derived exosomes (FB-Exos) and MSC-derived exosomes (MSC-Exos) (left); 5 downregulated microRNAs and 14 upregulated microRNAs are highlighted (right, n = 3 samples per group). The miR-125a-5p and miR-125b-5p levels in FB-Exos and MSC-Exos were quantified via quantitative reverse transcription polymerase chain reaction (RT-qPCR) and normalized to external control cel-miR-39 and internal control *U6*, respectively (bottom, *n* = 5 independent experiments; ^**^P < 0.01). (**B**) Experimental strategy in a mouse model of myocardial I/R injury. Mice underwent left anterior descending (LAD) coronary artery occlusion for 60 min, followed by reperfusion. Intramyocardial injection of 5 × 10^5^ MSCs (MSC group), 10 μg MSC-Exos (MSC-Exo group), or 20 nmol mmu-miR-125a-5p agomir (miR-125a-5p group) was performed at the onset of reperfusion. Administration of sterile phosphate buffer saline (PBS) (I/R control group) or 20 nmol negative control (NC) agomir (NC agomir group) in myocardial I/R mice was used as a corresponding control, and a sham control (Sham group) was also applied. Echocardiography and heart tissue collection were performed within 28 days post-cardiac I/R. (**C**) miR-125a-5p expression in the border zone of I/R hearts (*n* = 3-4 mice per group; ^*^P < 0.05, ^**^P < 0.01, I/R control group versus Sham group; ^&&^*P* < 0.01, miR-125a-5p group versus NC agomir group). (**D**) Kaplan-Meier curve showing mortality after myocardial I/R (*n* = 16-23 mice per group;^ *^P < 0.05 versus I/R control group; ^&^*P* < 0.05 versus NC agomir group). (**E-G**) Echocardiography for the assessment of cardiac function, including (E) left ventricular (LV) ejection fractions (LVEF), (F) LV fractional shortening (LVFS), and (G) LV internal diameter at the end-systole (LVIDs) at day 14 and 28 post-myocardial I/R. (H) Heart to body weight ratio 28 days after myocardial I/R. (I) The fibrotic area (%) of the mouse heart at day 28 post-myocardial I/R was calculated via dividing the scar area (black arrowheads) by the total LV area in five Masson's trichrome-stained slices from the apex to base. (**J**) Pie charts of the severity of fibrosis. Mild, moderate, and severe fibrosis were defined as a scar size < 25%, between 25% and 45%, and > 45% of the total LV area, respectively (*n* = 7-11 mice per group). (**K**) Wheat germ agglutinin (WGA) staining in representative cardiac sections (left) and quantification (right) in the border zone on day 28 post-cardiac I/R. Scale bar: 30 μm (*n* = 6 mice per group; ^*^P < 0.05 and ^**^P < 0.01). Statistical analysis was performed using one-way ANOVA followed by Tukey's post hoc test in (A, H, I, and K), two-way ANOVA followed by Tukey's post hoc test in (C and E-G), and log-rank test in (D).

We next compared the microRNA expression signatures of MSC-Exos and mouse cardiac fibroblast-derived exosomes (FB-Exos), as, to the best of our knowledge, no previous study has reported that the cardiac FB-Exos affect the regulation of macrophage polarization. Our results suggested that the levels of 14 microRNAs were increased in MSC-Exos, with two candidate microRNAs (miR-125a-5p and miR-125b-5p) being most significantly upregulated through microRNA sequencing (**Figure 1A**), both of which have been previously reported to be enriched in M2 macrophages 12. Furthermore, miR-125a-5p was detected as being more highly expressed than miR-125b-5p in MSC-Exos (**Figure 1A**) and was not enriched in exosomes secreted by other types of cardiac cells (cardiomyocytes, endothelial cells, and macrophages) (**Figure S3A**). Notably, the endogenous miR-125a-5p level was detected and found to be significantly decreased in response to transforming growth factor-beta1 (TGF-β1), hypoxia, and lipopolysaccharide (LPS) treatment in fibroblasts, endothelial cells, and macrophages, respectively, but not oxygen-glucose deprivation/recovery (OGD/R)-stimulated cardiomyocytes (**Figure S3B**). We then explored whether the agomir of MSC-Exo-enriched miR-125a-5p can play the same role as MSC or MSC-Exo transplantation in the context of myocardial I/R injury. We first confirmed that the dose of intramyocardial injection of miR-125a-5p agomir in the subsequent rodent study was 20 nmol because it was sufficient to significantly improve cardiac function (**Figure S4A-S4C**). Moreover, as 5 × 10^5^ MSCs are sufficient to protect the I/R myocardium (**Figure S4D-S4F**) 21, and considering that the MSC-Exos extracted from 5 × 10^5^ MSCs (cultured for 48 h) have a mass of approximately 10 μg, we then injected 5 × 10^5^ MSCs, 10 μg MSC-Exos, or 20 nmol miR-125a-5p agomir into the border zone at the onset of reperfusion (**Figure 1B**). Our results demonstrated that with the development of I/R, a progressive decrease in miR-125a-5p expression was observed in the I/R control group compared with that of the Sham group. Moreover, the hearts that received miR-125a-5p agomir showed a significant increase in miR-125a-5p level in the border zone, which persisted for up to 28 days after myocardial I/R (**Figure 1C**). Of note, no significant difference in mortality was observed between the three treatments (MSC, MSC-Exo, and miR-125a-5p agomir), although there was a notable improvement in survival compared with their corresponding control groups (**Figure 1D**).

The impact of MSC, MSC-Exo, or miR-125a-5p agomir treatment on cardiac function was next investigated by echocardiography on day 14 and 28 post-cardiac I/R. The I/R control and negative control (NC) agomir groups showed dramatic reductions in cardiac function, including decreases in left ventricular (LV) ejection fractions (LVEF) and LV fractional shortening (LVFS), and elevation in LV internal diameter at the end-systole (LVIDs), and all three treatments significantly prevented a decline in cardiac function on day 28 post-myocardial I/R (**Figure 1E-1G** and **Table S4**). We next sought to determine the effect of MSCs, MSC-Exos, and miR-125a-5p agomir on I/R-induced myocardial remodeling. On day 28 post-cardiac I/R, adverse LV remodeling was visible, supported by a significant increase in the heart to body weight ratio, ventricular fibrosis, and cross-sectional area of cardiomyocytes from the border zone of I/R hearts, all of which were reversed after three treatments (**Figure 1H-1K**). Furthermore, reduced infarction was observed 3 days after myocardial I/R in the hearts of the MSC, MSC-Exo, and miR-125a-5p groups (**Figure S4G**).

To further determine the indispensable role of miR-125a-5p in MSCs and MSC-Exos in the I/R myocardium, MSCs were transfected with miR-125a-5p antagomir to eliminate the expression of miR-125a-5p in cells (MSC^miR125a-anta^) and corresponding isolated exosomes (MSC^miR125a-anta^-Exo) (**Figure S5A**). An enlarged infarct was visible in the MSC^miR125a-anta^ and MSC^miR125a-anta^-Exo heart on day 3 post-myocardial I/R compared with that of the corresponding control heart (**Figure S5B**). Consistently, miR-125a-5p antagomir treatment partially reversed the improved cardiac function in the MSC-treated and the MSC-Exo-treated mice on day 14 and 28 (**Figure S5C-S5H**). In addition, none of the three treatments affected cardiac function in the sham-operated mice (**Figure S6A-S6C**). Taken together, these findings reveal that as one of the potential active ingredients of MSC-Exos, treatment with miR-125a-5p agomir showed a significant improvement in cardiac function and reduction in cardiac remodeling, similar to that observed with MSC or MSC-Exo administration in mice with myocardial I/R.

### miR-125a-5p agomir treatment in the mouse I/R heart attenuates cardiomyocyte apoptosis, fibroblast proliferation, and cardiac inflammation and increases angiogenesis

**Figure 2:**
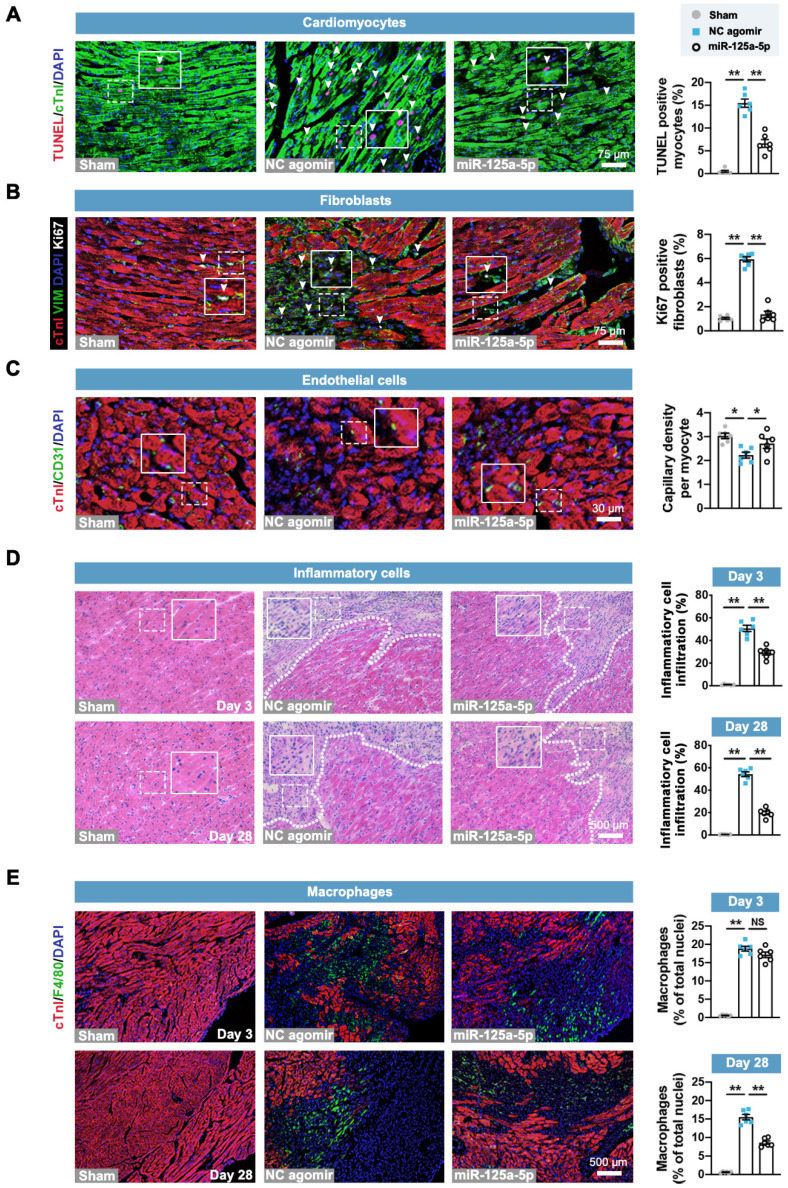
** Treatment of miR-125a-5p agomir inhibits cardiomyocyte apoptosis, fibroblast proliferation, and inflammatory response, and increases angiogenesis in mouse I/R myocardium.** Sections of hearts were collected from Sham, NC agomir, and miR-125a-5p animals 3 or 28 days after myocardial I/R induction. (**A**) Quantitative analysis of terminal deoxynucleotidyl transferase dUTP nick end labeling (TUNEL) and cardiac troponin I (cTnI) staining to evaluate cardiomyocyte apoptosis (TUNEL positive, white arrowheads) in the border zone of the infarct on day 3 post-cardiac I/R; frames show higher-magnification images of the area outlined by white dashed lines. cTnI staining was used as a cardiomyocyte marker, and 4',6-diamidino-2-phenylindole (DAPI) staining was applied to locate the nuclei. Scale bar: 75 μm. (**B**) Representative images (left) and quantitative assessment (right) of vimentin (fibroblast marker) and Ki67 (proliferation-related marker) positive cells (white arrowheads) for the border zone on day 28 post-cardiac I/R. Scale bar: 75 μm. (**C**) Representative images (left) and quantitative assessment (right) of CD31 staining (endothelial marker) for the border zone on day 28. Scale bars: 30 μm. (**D**) Representative images of hematoxylin and eosin (H&E) staining (left) and quantification of inflammatory cell infiltration (right: white dotted in H&E staining) in the Sham hearts or the border zone of I/R hearts on day 3 (upper panel) and 28 (lower panel). Scale bar: 500 μm. (**E**) Representative images and quantitative assessment of F4/80 staining (macrophage marker) of the border zone on day 3 (upper panel) and 28 (lower panel) post-myocardial I/R. Scale bars: 500 μm (*n* = 6 mice per group). Statistical analysis was performed using one-way ANOVA followed by Tukey's post hoc test. ^*^P < 0.05 and ^**^P < 0.01, NS: No significance.

Next, we investigated the roles of miR-125a-5p agomir in the key pathological processes related to cardiac remodeling post-I/R, including cardiomyocyte apoptosis, fibroblast proliferation, angiogenesis, and inflammation (**Figure 2A-2D**) 22. Immunostaining analysis revealed that cardiomyocyte apoptosis and fibroblast proliferation of the myocardium in the border zone of the infarct were significantly inhibited in the miR-125a-5p mice compared to the NC agomir mice (**Figure 2A** and** 2B**). Congruously, analysis of myocardial RNA from the border zone of the heart demonstrated that the increased expression of cardiac remodeling-related genes induced by I/R injury was significantly alleviated by miR-125a-5p agomir treatment (**Figure S7A** and** S7B**). Additionally, a significant increase in vascular density was observed in the border zone of miR-125a-5p agomir-treated I/R myocardium (**Figure 2C**). Hematoxylin and eosin (H&E) staining suggested that the infiltration of inflammatory cells in the border zone was reduced in mice that received miR-125a-5p agomir on both day 3 and day 28 post-cardiac I/R (**Figure 2D**), which was accompanied by a reduction in interleukin (IL)-6 (pro-inflammatory cytokine) and an increase in IL-10 (anti-inflammatory) levels in both the myocardium and serum (**Figure S7C** and **S7D**). We next determined the role of miR-125a-5p agomir in macrophage infiltration to the I/R heart. The results demonstrated a significant reduction in the infiltration of macrophages in the border zone on day 28 (**Figure 2E**). However, the macrophage infiltration on day 3 was not significantly altered between the NC agomir and miR-125a-5p agomir-treated mice (**Figure 2E**). Collectively, these results suggest that miR-125a-5p agomir delayed the pathological processes related to cardiac remodeling.

### miR-125a-5p directly regulates the function of stimulated macrophages, fibroblasts, and endothelial cells but not cardiomyocytes

**Figure 3:**
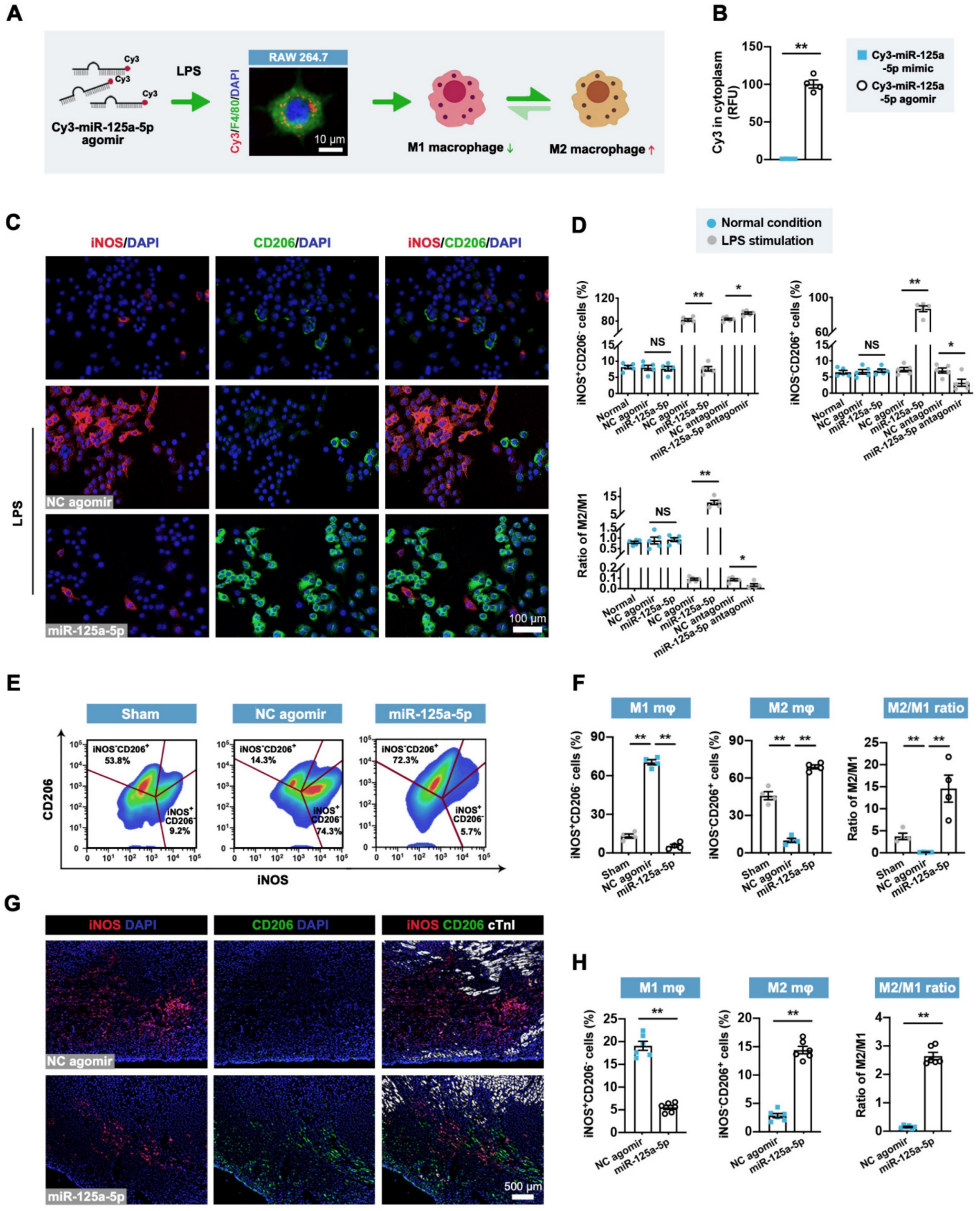
** miR-125a-5p mediates the conversion of pro-inflammatory M1 macrophages to an anti-inflammatory M2 phenotype in mouse I/R hearts.** (**A**) miR-125a-5p agomir was transfected into lipopolysaccharide (LPS)-stimulated (100 ng/mL) murine macrophage RAW 264.7 cells to switch the macrophage polarization from M1 to M2. The uptake of miR-125a-5p agomir in macrophages was confirmed by Cy3 fluorescence. Scale bar: 10 μm. (**B**) Quantitative assessment of Cy3 fluorescence intensity in the cytoplasm of the LPS-stimulated RAW 264.7 cells transfected with 100 nM Cy3-miR-125a-5p mimic or Cy3-miR-125a-5p agomir (*n* = 4 independent experiments). RFU: Relative fluorescence units. (**C**) Representative staining of inducible nitric oxide synthase (iNOS; M1 marker) and CD206 (M2 marker) in normal and LPS-stimulated RAW 264.7 cells transfected with 100 nM miR-125a-5p agomir or NC agomir. Scale bar: 100 μm. (**D**) Quantification of M1 and M2 percentages and the M2/M1 ratio in normal or LPS-stimulated RAW 264.7 macrophages transfected with miR-125a-5p agomir, NC agomir, miR-125a-5p antagomir, or NC antagomir (*n* = 5 independent experiments). (**E and F**) FCM analysis of macrophage polarization in mouse hearts on day 3 post-myocardial I/R. (E) Representative plot and (F) quantitative analysis of M1 macrophages (iNOS^+^CD206^-^) and M2 macrophages (iNOS^-^CD206^+^) in the Sham, NC agomir, and miR-125a-5p mice (*n* = 4 mice per group). (**G and H**) Macrophage polarization in the border zone was further confirmed via immunofluorescent analysis on day 3. (G) Representative images and (H) quantitative assessment of iNOS and CD206 staining. Scale bar: 500 μm (*n* = 6 mice per group). Statistical analysis was performed using Student's *t*-test in (B, D, and H) and one-way ANOVA followed by Tukey's post hoc test in (F).^ *^P < 0.05 and ^**^P < 0.01, NS: No significance.

Considering the reduction of inflammatory responses after miR-125a-5p agomir treatment, as well as the high miR-125a-5p level in M2 macrophages 12, we next investigated the effect of miR-125a-5p agomir on macrophage polarization* in vitro*. Successful transfection of miR-125a-5p agomir in murine macrophage RAW 264.7 cells was confirmed by Cy3 fluorescence (labeled at the 5' end of the miR-125a-5p agomir) (**Figure 3A**). Due to the high transfection efficiency caused by the 2'OMe and 5'chol modification, the miR-125a-5p agomir was more abundantly enriched in the cytoplasm than the miR-125a-5p mimic (**Figure 3B**). Although miR-125a-5p agomir had no significant effect on macrophage polarization under normal conditions, treatment with LPS-stimulated RAW 264.7 cells with miR-125a-5p agomir resulted in a significant decline in the M1 macrophage phenotype and a significant increase in the M2 phenotype, ultimately leading to an elevated M2/M1 ratio compared to that observed with the NC agomir treatment (**Figure 3C** and **3D**). Moreover, the application of miR-125a-5p antagomir further abolished the effects of endogenous miR-125a-5p on macrophage polarization (**Figure 3D**). To further verify the role of the miR-125a-5p agomir in macrophage polarization *in vivo*, an FCM-based approach was used on day 3 post-myocardial I/R (**Figure 3E**). We observed a significant increase in the M1 phenotype and a significant reduction in the M2/M1 ratio in the I/R myocardium, both of which were reversed by miR-125a-5p agomir treatment (**Figure 3F**). Immunostaining of the border zone myocardium indicated that miR-125a-5p agomir also promoted the regional conversion of macrophage polarization from M1 to M2 3 days after cardiac I/R (**Figure 3G** and **3H**). Conformably, in the border zone of the I/R heart, prominent M1 macrophage polarization was determined by marked upregulation in the mRNA expression of M1 markers (inducible nitric oxide synthase [iNOS]) and pro-inflammatory cytokines (IL-6, IL-1β, and tumor necrosis factor-alpha [TNF-α]) (**Figure S8A**). The application of miR-125a-5p agomir alleviated all of the alterations induced by myocardial I/R and mediated a significant increase in the mRNA expression of M2 markers (CD206) and anti-inflammatory cytokines (IL-10, arginase 1 [Arg1], and TGF-β) (**Figure S8A** and **S8B**). To further confirm the dominance of macrophages, we systemically depleted macrophages in the mouse via intraperitoneal injection of dichloromethylene diphosphonate liposomes (Cl_2_MDP-Lipo) (**Figure S9A**). As expected, after reducing macrophage involvement, miR-125a-5p agomir lost most of its cardioprotective effects, such as its ability to reduce cardiomyocyte apoptosis and improve cardiac function (**Figure S9B and S9C**). Notably, macrophage depletion alone had no significant effect on the I/R heart (**Figure S9A-S9C**).

**Figure 4:**
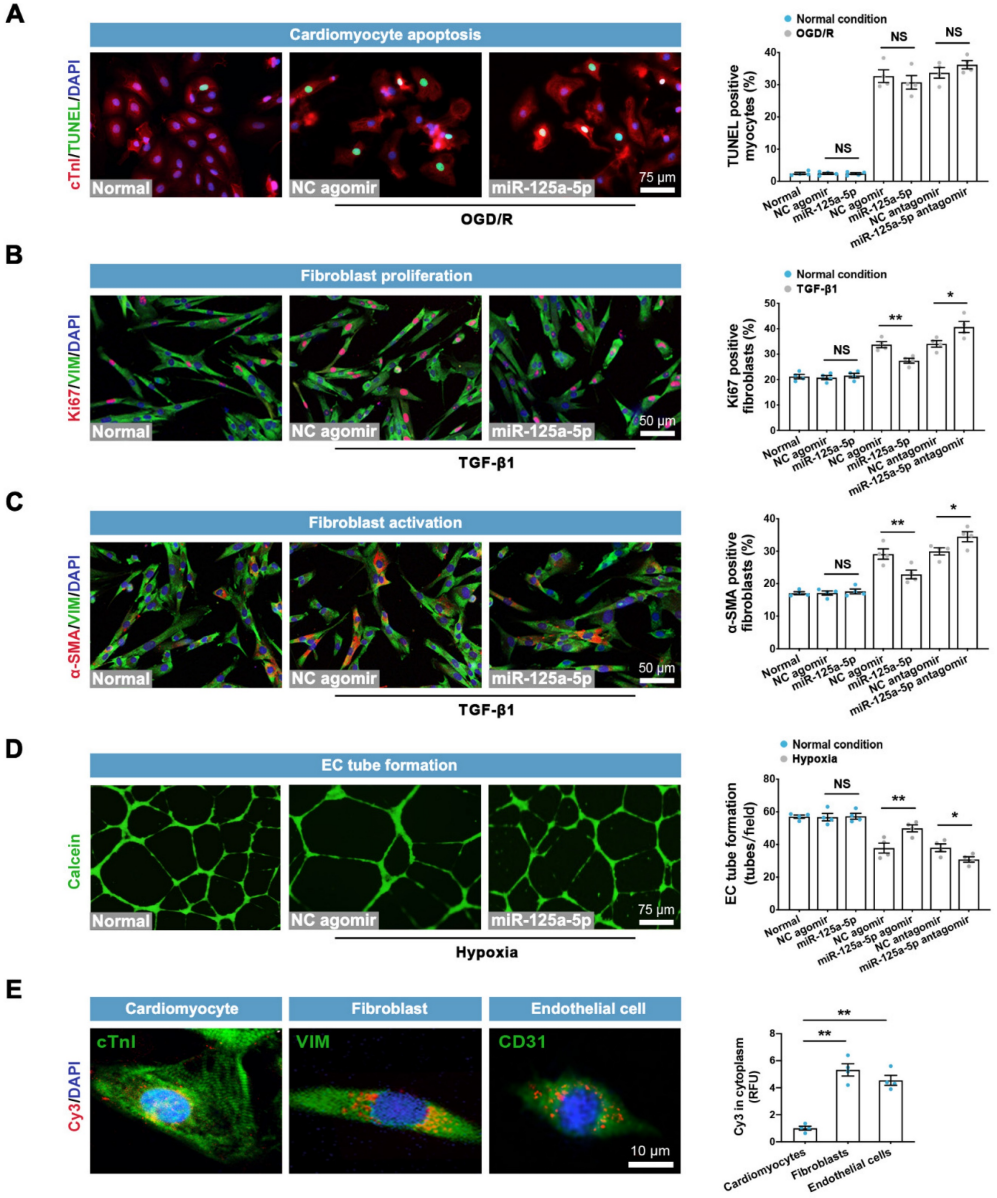
** Effects of miR-125a-5p agomir on cardiomyocyte apoptosis, fibroblast proliferation and activation, and EC tube formation *in vitro*.** (**A**) Representative images and quantitative assessment of TUNEL-positive primary neonatal mouse cardiomyocytes in normal, normal + NC agomir, normal + miR-125a-5p agomir, oxygen-glucose deprivation/recovery (OGD/R) + NC agomir, OGD/R + miR-125a-5p agomir, OGD/R + NC antagomir, and OGD/R + miR-125a-5p antagomir groups. Scale bar: 75 μm. (**B**) Representative images and quantitative analysis of Ki67 positive cardiac fibroblasts in normal, normal + NC agomir, normal + miR-125a-5p agomir, TGF-β1 + NC agomir, TGF-β1 + miR-125a-5p agomir, TGF-β1 + NC antagomir, and TGF-β1 + miR-125a-5p antagomir groups. Scale bar: 50 μm. (**C**) Representative images and quantification of α-smooth muscle actin (α-SMA) positive fibroblasts. Scale bars: 50 μm. (**D**) Representative images of calcein-labeled endothelial cells (ECs) in normal, normal + NC agomir, normal + miR-125a-5p agomir, hypoxia + NC agomir, hypoxia + miR-125a-5p agomir, hypoxia + NC antagomir, and hypoxia + miR-125a-5p antagomir groups; tube formation was quantified by determining the number of tubes per field. Scale bar: 75 μm. (**E**) The uptake of miR-125a-5p agomir in cardiomyocytes, fibroblasts, ECs, and macrophages was investigated by Cy3 fluorescence. The relative fluorescence unit (RFU) was assessed and normalized to cardiomyocytes. Scale bar: 10 μm (*n* = 4 independent experiments). Statistical analysis was performed using Student's *t*-test in (A-D) and one-way ANOVA followed by Tukey's post hoc test in (E).^ *^P < 0.05 and ^**^P < 0.01, NS: No significance.

We next determined the direct roles of miR-125a-5p on other cardiac cells* in vitro*. The results showed that neither miR-125a-5p agomir nor antagomir had a significant impact on apoptosis in normal or OGD/R-insulted cardiomyocytes (**Figure 4A**). Although miR-125a-5p did not affect cardiac fibroblasts under normal conditions, it inhibited the proliferation and activation of fibroblasts in the presence of TGF-β1, which was abolished by miR-125a-5p antagomir (**Figure 4B** and** 4C**). Moreover, miR-125a-5p significantly promoted the tube-forming activity of endothelial cells (ECs) under hypoxic but not normal conditions, and inhibition of miR-125a-5p abrogated these effects (**Figure 4D**). Further experiments showed that despite partial uptake of exogenous miR-125a-5p in cardiomyocytes, miR-125a-5p agomir was predominantly taken up by fibroblasts, ECs, and macrophages (**Figures 4E** and** 3A**). The above findings reveal that in addition to regulating the polarization of M2 macrophages, miR-125a-5p acts directly on cardiac fibroblasts and ECs to modulate their functions.

### Downstream mechanisms of miR-125a-5p in regulating macrophage, fibroblast, and EC functions

**Figure 5:**
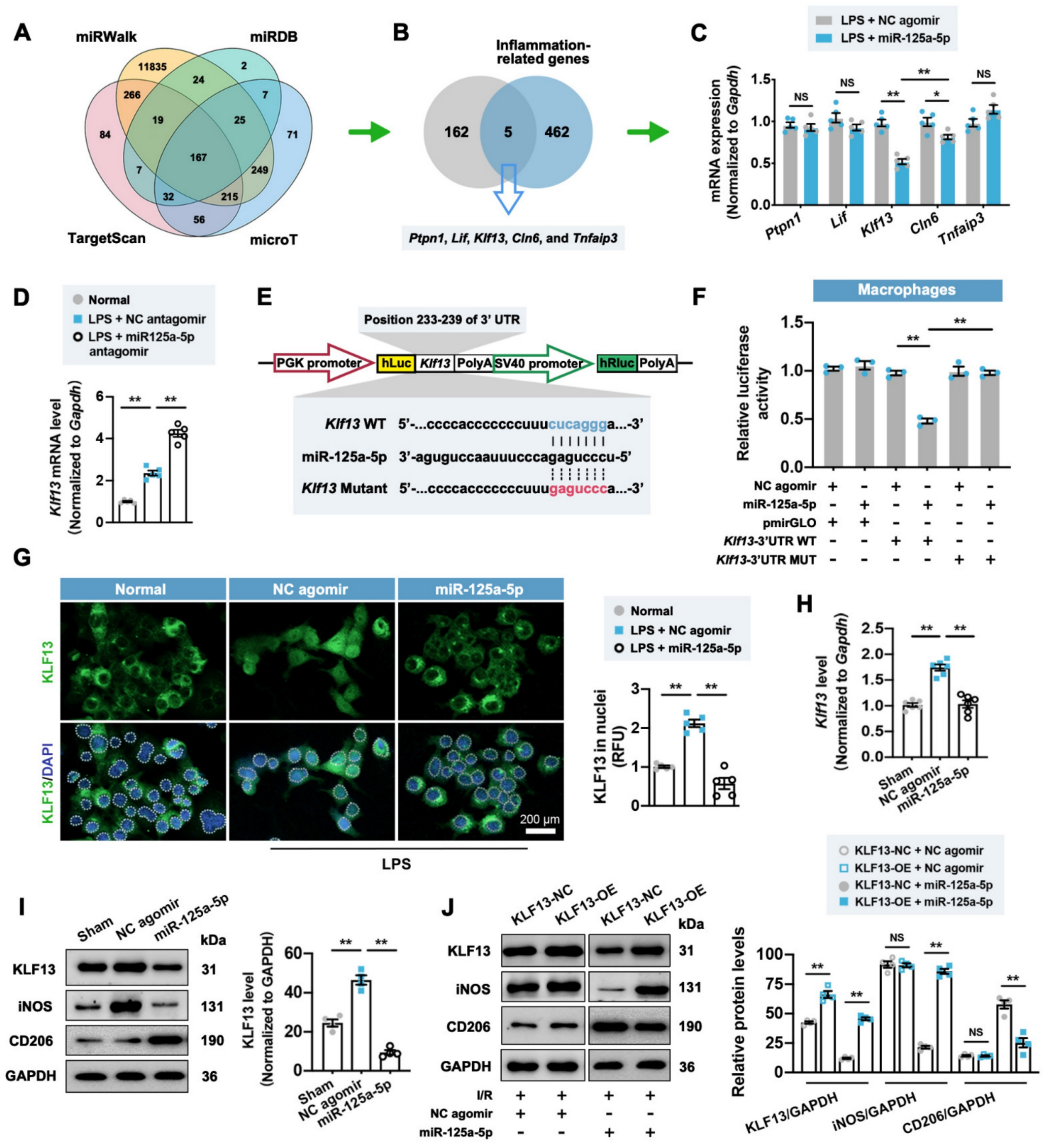
** miR-125a-5p regulates macrophage polarization in the I/R myocardium of mice by repressing the expression and nuclear translocation of Krüppel-like factor 13 (KLF13).** (**A**) Four online databases (miRWalk, miRDB, TargetScan, and microT) were used to predict 167 candidate genes that may be targeted by miR-125a-5p. (**B**) The 167 miR-125a-5p target genes predicted from online databases and 467 inflammation-related genes predicted from Disgenet were intersected to obtain five candidate genes. (**C**) The mRNA expression levels of *ptpn1*, *lif*, *Klf13*, *cln6*, and *tnfaip3* were detected by RT-qPCR in LPS-stimulated RAW 264.7 cells transfected with miR-125a-5p agomir or NC agomir. (**D**) The mRNA expression level of *Klf13* was detected in normal RAW 264.7 cells and LPS-stimulated cells transfected with miR-125a-5p antagomir or NC antagomir (*n* = 5 independent experiments). (**E**) A wild type (WT) or mutant dual-luciferase reporter plasmid was constructed according to the predicted binding sequence in 3' UTR of *Klf13* (blue) or mutant sequence (red). (**F**) Luciferase activity was determined in RAW 264.7 transfected with WT or mutant reporter plasmids and 100 nM miR-125a-5p agomir or NC agomir (*n* = 3 independent experiments). (**G**) Representative images (left) and quantitative assessment (right) of KLF13 staining in the nuclei (white dotted) of normal RAW 264.7 cells and LPS-stimulated cells transfected with 100 nM miR-125a-5p agomir or NC agomir. Scale bar: 200 μm (*n* = 5 independent experiments). (**H**) The mRNA expression level of *Klf13* was detected by RT-qPCR in the Sham, NC agomir, and miR-125a-5p mice 3 days after myocardial I/R (*n* = 6 mice per group). (**I**) The protein expression of KLF13 was assessed by western blotting. (**J**) KLF13 overexpression (KLF13-OE) or negative control (KLF13-NC) lentiviruses were injected intramyocardially 7 days prior to myocardial I/R surgery and agomir treatment, and the representative immunoblots and quantification for KLF13, iNOS, and CD206 proteins on day 3 post-cardiac I/R are shown (*n* = 4 mice per group). Statistical analysis was performed using Student's *t*-test in (C and J) and one-way ANOVA followed by Tukey's post hoc test in (D, F, H, and I). ^*^P < 0.05 and ^**^P < 0.01, NS: No significance.

It is well established that microRNA can bind one or more mRNA molecules to induce their rapid degradation, thus decreasing the levels of protein translated from these mRNAs 23. We therefore adopted a database-based prediction method 23 to investigate the downstream genes targeted by miR-125a-5p in this model. Through the pairing between the miR-125a-5p seed sequence and the complementary region within mRNAs, we recognized 167 target genes (**Figure 5A**). These 167 genes were intersected with 467 predicted inflammation-related genes to obtain five candidate genes, including *ptpn1*, *lif*, *Klf13*, *cln6*, and *tnfaip3* (**Figure 5B**). Among them, *Klf13* displayed the most prominent reduction in the stimulated RAW 264.7 macrophages treated with miR-125a-5p agomir (**Figure 5C**), while inhibition of miR-125a-5p significantly reversed *Klf13* downregulation (**Figure 5D**). To further determine the direct interaction between miR-125a-5p and *Klf13*, we performed a luciferase activity assay using a dual-luciferase reporter plasmid carrying the predicted miR-125a-5p binding sites in the 3' UTR of *Klf13* (**Figure 5E**). The results demonstrated that the luciferase activity of the* Klf13* wild-type (WT) vector was significantly decreased by miR-125a-5p agomir treatment, whereas mutation of the binding sites abolished this inhibitory effect in RAW 264.7 cells (**Figure 5F**). Importantly, a significant inhibition in nuclear translocation of Krüppel-like factor 13 (KLF13) was observed in the LPS + miR-125a-5p agomir-treated RAW 264.7 macrophages (**Figure 5G**). In line with the *in vitro* observations, the expression of *Klf13* and its encoded protein KLF13 in the miR-125a-5p agomir-treated I/R myocardium was significantly downregulated compared to that of the NC agomir-treated myocardium (**Figure 5H** and **5I**). Moreover, overexpression of KLF13 by lentiviruses significantly promoted M1 macrophage marker and reduced M2 marker expression in the I/R myocardium treated with miR-125a-5p agomir but not NC agomir (**Figure 5J**). We also investigated the potential signaling cascades in the miR-125a-5p agomir-treated I/R myocardium (**Figure S10**). Four canonical stress-activated kinases, including Akt and mitogen-activated protein kinase (MAPK) family kinases (extracellular-regulated kinase 1/2 [ERK1/2], P38, and c-Jun N-terminal kinase [JNK]) were examined, which have been implicated to play an important role in cardiac ischemic preconditioning and may be involved in the regulation of cardiac remodeling in the I/R myocardium 24, 25. The results suggested that myocardial I/R significantly elevated the phosphorylation of the four kinases, but only the phosphorylation of ERK1/2 (p-ERK1/2) was inhibited in the miR-125a-5p group on day 3 post-IR (**Figure S10A**). Furthermore, LY2828360-induced activation of ERK1/2 drastically reversed macrophage polarization in the miR-125a-5p agomir-treated I/R myocardium (**Figure S10B**).

We also investigated the mechanism by which miR-125a-5p regulates fibroblast proliferation and activation. To this end, the 167 predicted miR-125a-5p target genes were intersected with 297 cardiac fibrosis-related genes to obtain four candidate genes, namely *Rit1*, *Nlrc5*, *Tgfbr1*, and *Sirt7* (**Figure S11A**). The downregulation of *Tgfbr1* was most pronounced in miR-125a-5p agomir-treated cardiac fibroblasts (**Figure S11B**), and inhibition of miR-125a-5p reversed the decrease in *Tgfbr1* (**Figure S11C**). The results of the luciferase activity assay confirmed the direct interaction of miR-125a-5p with sites 2243-2249 of the 3' UTR of *Tgfbr1* (**Figure S11D** and **S11E**). Importantly, miR-125a-5p reduced TGF-β receptor-1 (TGFBR1) expression in both normal conditions and in TGF-β1-treated fibroblasts (**Figure S11F**), while TGFBR1 overexpression eliminated the decreased proliferation and activation of fibroblasts induced by miR-125a-5p in the presence of TGF-β1, but not under normal conditions (**Figure S11G** and **S11H**). The *in vivo* rescue data also confirmed that miR-125a-5p agomir-mediated decrease in interstitial fibrosis was abolished by TGFBR1 overexpression in mouse I/R myocardium (**Figure S11I**). Additionally, the *Daam1* gene was screened as a potential downstream target for miR-125a-5p to regulate EC function (**Figure S12A-S12E**), and declined expression of dishevelled associated activator of morphogenesis 1 (DAAM1) was observed in miR-125a-5p-treated ECs under normal and hypoxic conditions (**Figure S12F**). DAAM1 overexpression also abrogated the promotion of EC functions by miR-125a-5p in hypoxia-treated ECs* in vitro* and I/R myocardium *in vivo* (**Figure S12G** and** S12H**). Of note, overexpression of KLF13, TGFBR1, or DAAM1 partially abolished the cardiac function-improving effect of miR-125a-5p in myocardial I/R mice (**Figure S13A-S13F**). These findings indicate that miR-125a-5p promotes macrophage polarization by targeting *Klf13* and inactivating the ERK1/2 pathway, inhibits fibroblast proliferation and activation through targeting *Tgfbr1*, and increases angiogenesis via interacting with* Daam1*.

### Application of miR-125a-5p agomir improves global and regional myocardial performance and reduces cardiac remodeling in a porcine I/R model

**Figure 6:**
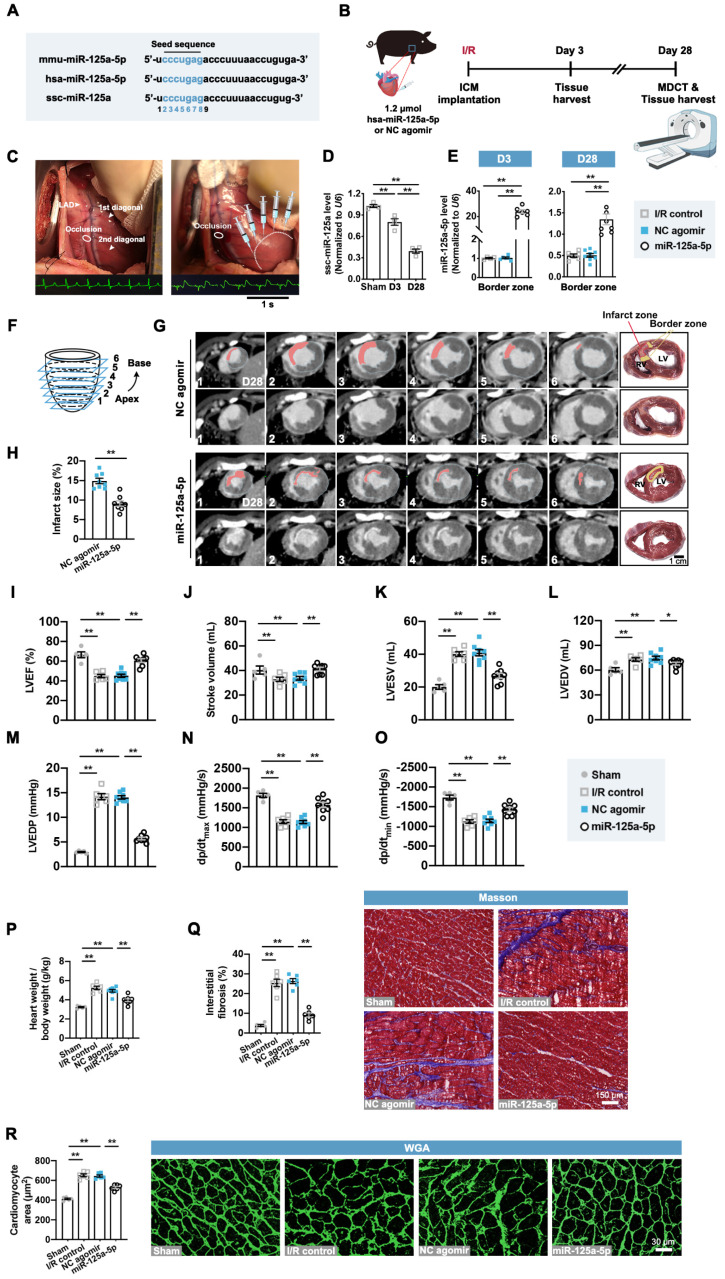
** miR-125a-5p enhances myocardial function and limits cardiac remodeling in a myocardial I/R porcine model.** (**A**) Mature sequences of mmu-miR-125a-5p, hsa-miR-125a-5p, and ssc-miR-125a conserved in mouse, human, and swine. The microRNA seed sequences are shown in blue. (**B**) Experimental strategy in a porcine model with myocardial I/R. Swine underwent LAD occlusion for 60 min, followed by reperfusion and intramyocardial injection of 1.2 μmol hsa-miR-125a-5p agomir (miR-125a-5p group) or equal amounts of NC agomir (NC agomir group). Sham control (Sham group) and PBS administered swine (I/R control group) were also applied. For short-term assessment, swine were sacrificed on day 3 post-myocardial I/R; for long-term assessment, swine were scanned with cardiac multi-detector computed tomography (MDCT) to assess cardiac function and infarct size on day 28 post-myocardial I/R; heart tissues were then collected for further analyses. (**C**) Representative photographs of the sites of occlusion and injection taken during the porcine myocardial I/R surgery. After the occlusion located between the two diagonal branches on the LAD and the subsequent reperfusion, injections were performed intramyocardially into the border zone (white dotted). Normal electrocardiogram (ECG) before the occlusion and ECG with ST elevation during the occlusion are shown below. (**D**) Expression of miR-125a in the Sham hearts and border zone of I/R hearts (*n* = 4 swine per group). (**E**) Quantification of the miR-125a-5p level (exogenous) in the I/R control, the NC agomir, or miR-125a-5p agomir-treated myocardium on days 3 and 28 post-cardiac I/R (*n* = 4-8 swine per group). (**F**) Diagram of delayed enhancement (de)-MDCT slices from the apex to base (1 to 6). (**G**) Cardiac short-axis de-MDCT images (from 1-6) of representative swine injected with NC agomir or miR-125a-5p agomir on day 28 post-myocardial I/R. The infarct area is depicted with a computer-generated mask in red (upper panels), and the corresponding original images are shown (lower panels). Myocardial slice anatomy of each heart showing the infarct zone (red) and border zone (yellow). Scale bar: 1 cm. RV: right ventricle, LV: Left ventricle. (**H**) The infarct size (%) was calculated by dividing the infarct area by the total LV area labeled in (G). (**I to L**) Cardiac function, including (I) LVEF, (J) stroke volume, (K) LV end-systolic volume (LVESV), and (L) LV end-diastolic volume (LVEDV), of the Sham, I/R control, or the NC agomir or miR-125a-5p agomir-treated swine was assessed by MDCT 28 days after myocardial I/R. (**M-O**) Hemodynamic measurements of (M) LV end-diastolic pressure (LVEDP), (N) peak contraction velocity (dp/dt_max_), and (O) peak relaxation velocity (dp/dt_min_) were made at day 28 post-myocardial I/R. (**P**) The heart to body weight ratio 28 days after myocardial I/R (*n* = 5-8 swine per group). (**Q**) Quantitative assessment (left) and representative images (right) of Masson's trichrome staining in the border zone at day 28 post-myocardial I/R. Scale bar: 150 μm. (**R**) Quantitative assessment (left) and representative images (right) of WGA staining in the border zone on day 28 post-myocardial I/R. Scale bar: 30 μm (*n* = 5-6 swine per group). Statistical analysis was performed by two-way ANOVA followed by Tukey's post hoc test in (D), one-way ANOVA followed by Tukey's post hoc test in (E and I-R), and Student's *t*-test in (H). ^*^P < 0.05 and ^**^P < 0.01.

We next investigated the role of the miR-125a-5p agomir in a porcine myocardial I/R model with promising translational potential. Consistent with the effects of miR-125a-5p in mice, the mature forms of miR-125a-5p in humans and miR-125a in swine are produced from the 5' arm of a hairpin precursor, despite a base lost in the *Sus scrofa* (ssc)-miR-125a (swine) (**Figure 6A**). Additionally, these three microRNAs share the same seed sequence, which is considered as the decisive factor for microRNA targeting patterns and effects (**Figure 6A**) 26. As there is increasing evidence that microRNAs can work across species 15, 27, 28, we sought to determine the roles of *Homo sapiens* (hsa)-miR-125a-5p agomir in swine myocardial I/R considering a better clinical translation. As shown in **Figure S14A-S14C**, administration of miR-125a-5p agomir consistently inhibited inflammatory infiltration and IL-6 levels, as well as increased IL-10 levels in the I/R myocardium on day 3. We further investigated the cardioprotective role of miR-125a-5p in the agomir-treated I/R myocardium (**Figure 6B** and** 6C**). As shown in **Figure 6D** and** 6E**, the expression of endogenous ssc-miR-125a was progressively decreased in the border zone of the I/R heart, and intramyocardial injection of miR-125a-5p agomir resulted in a significant elevation of miR-125a-5p levels that persisted for up to 28 days after cardiac I/R.

We then analyzed the infarct size and cardiac function though the multi-detector computed tomography (MDCT) images of six cross-sections (1 to 6 from apex to base) of hearts (**Figure 6F**). A transmural myocardial infarction invading the partial right ventricle was visible in the NC agomir swine, while a significant decrease in infarct proportion was observed in the myocardial I/R swine that received miR-125a-5p agomir (**Figure 6G** and** 6H**). Cardiac MDCT analysis suggested that myocardial I/R injury led to a significant decline in LVEF and stroke volume, as well as a significant increase in LV end-systolic volume (LVESV) and LV end-diastolic volume (LVEDV), and all of which were markedly improved after miR-125a-5p agomir administration in porcine hearts (**Figure 6I-6L** and **Table S5**). Hemodynamic analyses also showed that miR-125a-5p agomir treatment enhanced the functional performance of the I/R hearts (**Figure 6M-6O**). We next used a standard myocardial 17-segment model for the MDCT-based assessment of myocardial perfusion and wall motion to elaborate the regional cardiac function (**Figure S14D**). The results demonstrated that myocardial I/R injury primarily reduced the perfusion index and deteriorated the segmental motion in the middle and apex of the LAD supply area of the LV myocardium, while application of the miR-125a-5p agomir reversed this impaired regional heart function on day 28 (**Figure S14E** and **S14F**).

Consistent with the mouse data, miR-125a-5p agomir treatment markedly reversed the harmful cardiac remodeling induced by myocardial I/R, including reduction in the ratio of heart weight to body weight, myocardial fibrosis, and cardiomyocyte hypertrophy (**Figure 6P-6R**). In conclusion, these results reveal that miR-125a-5p agomir therapy can improve cardiac function and limits cardiac remodeling following myocardial I/R injury in swine.

### miR-125a-5p agomir delivery decreases myocardial I/R-induced remodeling-associated pathological processes in porcine hearts

**Figure 7:**
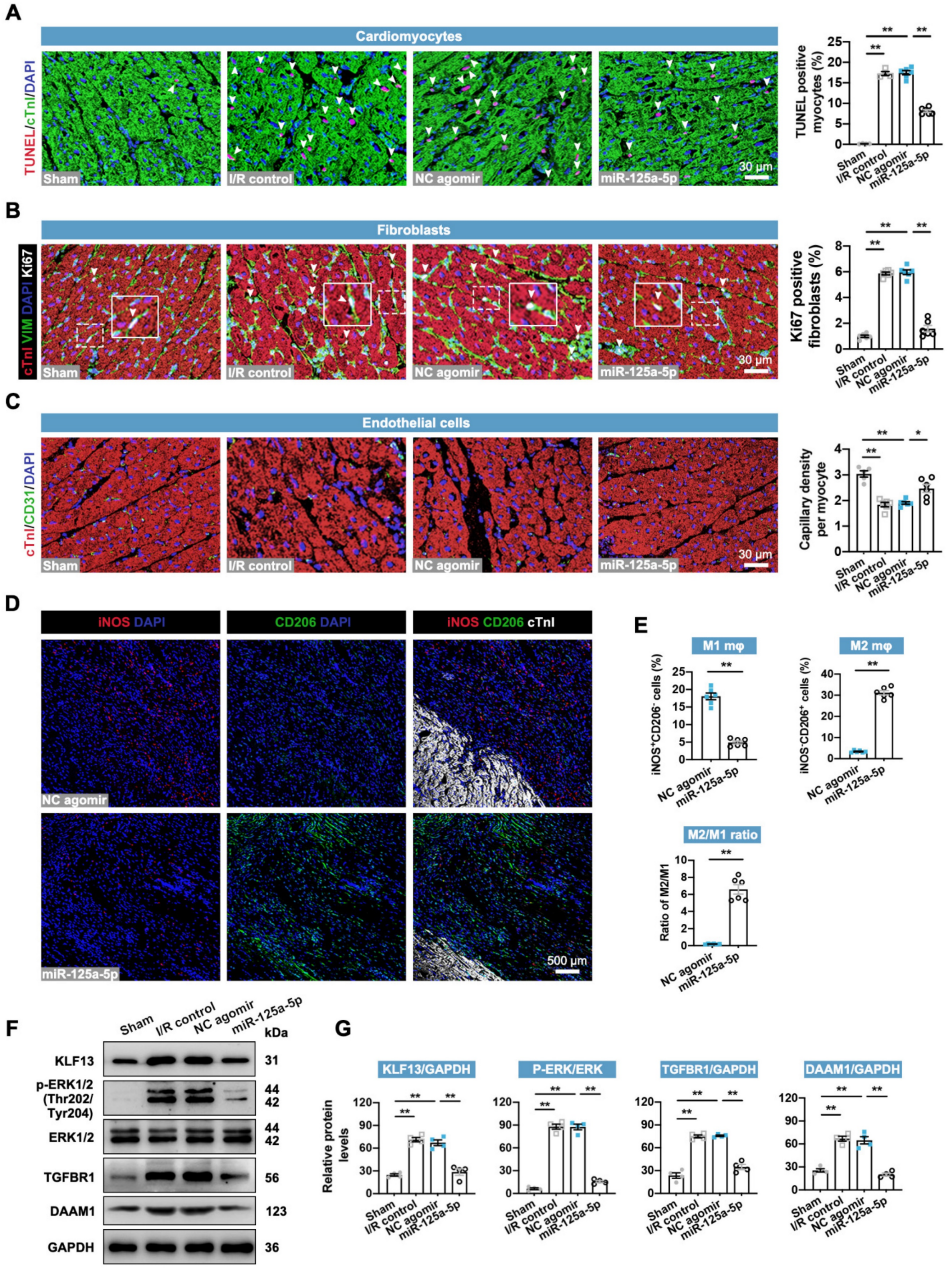
**miR-125a-5p therapy alleviates ventricular remodeling-associated pathological processes in porcine I/R hearts.** Sections of porcine hearts were collected from Sham, I/R control, NC agomir, and miR-125a-5p animals 3 or 28 days after myocardial I/R induction. (**A**) Representative images (left) and quantitative analysis (right) of TUNEL and cTnI staining to evaluate the percentages of TUNEL-positive cardiomyocytes (white arrowheads) in the border zone 3 days after cardiac I/R. Scale bar: 30 μm. (B) Representative images (left) and quantitative assessment (right) of vimentin and Ki67 positive cells (white arrowheads) in the border zone on day 28 post-myocardial I/R; frames showed higher-magnification images of the area outlined by white dashed lines. Scale bar: 30 μm. (**C**) Representative images (left) and quantitative assessment (right) of CD31 staining in the border zone on day 28. Scale bars: 30 μm. (**D and E**) On day 3 post-myocardial I/R, macrophage polarization in the myocardium was confirmed. (**D**) Representative staining and (**E**) quantitative assessment of M1 and M2 macrophages in the border zone. Scale bar: 500 μm (*n* = 5-6 swine per group). (**F and G**) Heart tissue from the border zone was collected for western blotting on day 3 post-myocardial I/R. (F) Representative immunoblots and (G) quantification of KLF13, p-ERK1/2, ERK1/2, TGFBR1, and DAAM1 proteins (*n* = 4 swine per group). Statistical analysis was performed using one-way ANOVA followed by Tukey's post hoc test in (A-C and G) and Student's *t*-test in (E). ^*^P < 0.05 and ^**^P < 0.01.

The regulatory roles of miR-125-5p agomir in cardiac cells in swine were then determined. As expected, the application of miR-125a-5p agomir inhibited the cardiac I/R-induced alterations in cardiomyocyte apoptosis, fibroblast proliferation, angiogenesis, inflammation, and cardiac remodeling-related gene levels (**Figure 7A-7C**, **Figure S14A**, and **Figure S15A**). However, no significant changes were observed in cardiomyocyte proliferation between the NC agomir and miR-125a-5p groups (**Figure S15B**). Furthermore, concordant with the immunostaining data in mice (**Figure 3G**), miR-125-5p agomir treatment significantly promoted the conversion of M1 to M2 in the border zone of I/R heart on day 3 and eventually led to a significant increase in M2/M1 ratio (**Figure 7D** and **7E**). We also verified the downstream mechanisms of miR-125a-5p on intramyocardial macrophage, fibroblast, and EC functions (**Figure 7F** and **7G**). Our results revealed a significant increase in KLF13 and p-ERK1/2 protein levels in porcine myocardial lysates following 3-day myocardial I/R injury, while injection of miR-125a-5p agomir significantly decreased these protein levels. Intramyocardial application of miR-125a-5p agomir also inhibited TGFBR1 and DAAM1 expression in the I/R heart. Collectively, these data indicate that miR-125a-5p agomir reduces cardiomyocyte apoptosis, fibroblast proliferation, and inflammation, and elevates angiogenesis and M2 macrophage polarization in porcine I/R myocardium with the involvement of KLF13, TGFBR1, and DAAM1 repression and ERK1/2 inactivation.

### Myocardial I/R swine receiving miR-125a-5p agomir show no alterations in arrhythmic occurrences and serum chemistries

**Figure 8:**
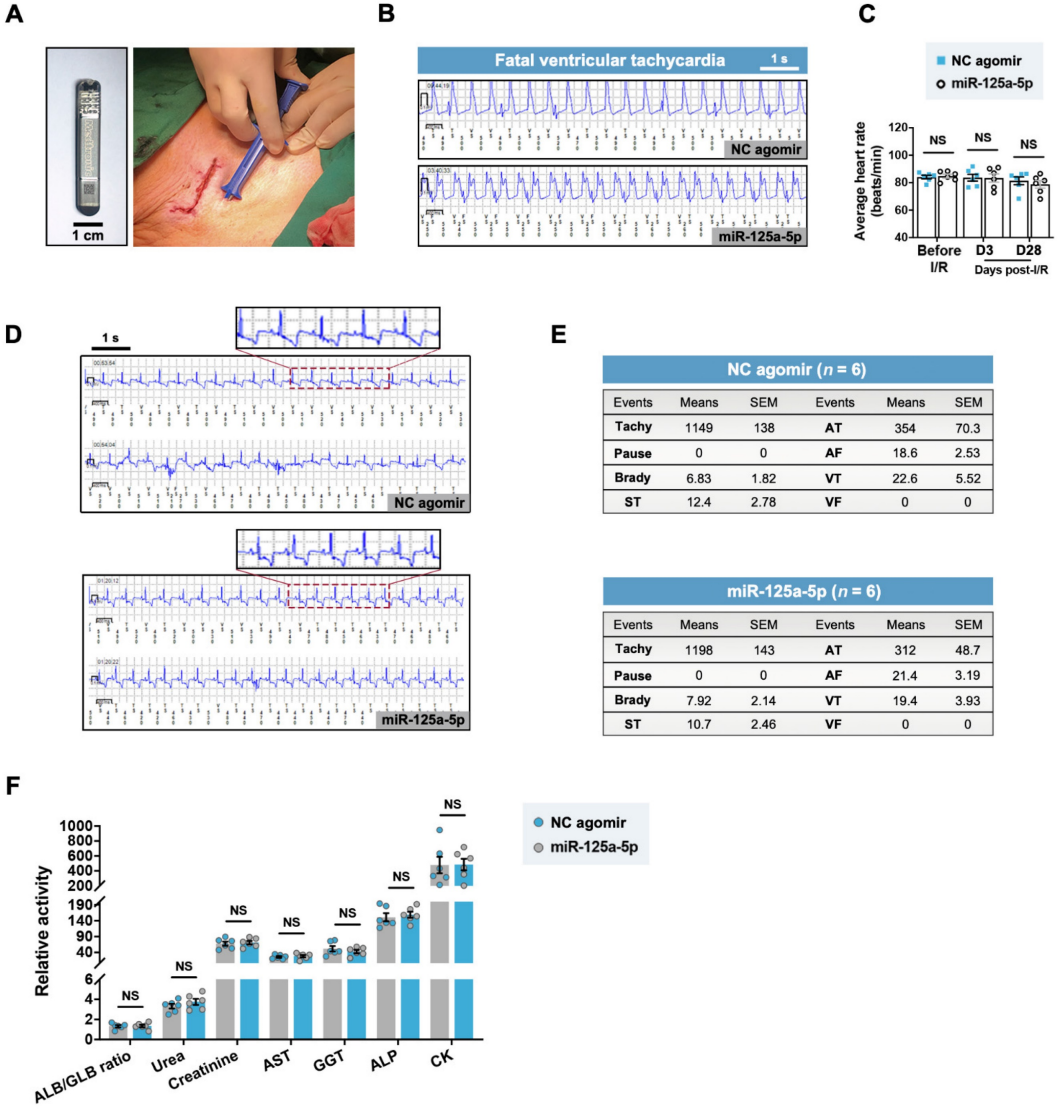
** miR-125a-5p agomir has no effect on the incidence of arrhythmia in myocardial I/R swine.** (**A**) After myocardial I/R surgery, an insertable cardiac monitor (ICM) device was implanted subcutaneously to continuously detect the electrical activity of the heart. A photo of the ICM device is shown in the left of the figure. Scale bar: 1 cm. (**B**) Representative electrocardiogram (ECG) episodes of sustained ventricular tachycardia (> 30 s) from the NC agomir swine that died on day 2 and the miR-125a-5p swine that died on day 3 post-myocardial I/R. (**C**) The mean heart rates at baseline, 3 days, and 28 days after cardiac I/R were recorded (*n* = 6 swine per group). (**D**) Representative ECG episodes (upper panels showing higher-magnification episodes of the area outlined) recorded by ICM in two randomly selected NC agomir and miR-125a-5p swine. Most ECGs were detected with a negative T wave, showing evidence of chronic myocardial ischemia 59. (**E**) During the 28-day observation period after myocardial I/R surgery, the number of arrhythmic events (Tachy: Tachycardia, Brady: Bradycardia, ST: Paroxysmal supraventricular tachycardia, AT: Atrial tachycardia, AF: Atrial fibrillation, VT: Paroxysmal ventricular tachycardia, VF: Ventricular fibrillation) in the NC agomir or miR-125a-5p agomir-treated swine is shown in the tables. The number of swine per group is indicated. (**F**) Analysis of serum chemistry relating to renal (ALB/GLB ratio, urea [mmol/L] and creatinine [μmol/L]), hepatic (AST [U/L], GGT [U/L], and ALP [U/L]), and cardiac indicators (CK [U/L]) (*n* = 6 swine per group). ALB: Albumin, GLB: Globulin, AST: Aspartate aminotransferase, GGT: γ-glutamyl transferase, ALP: Alkaline phosphatase, CK: Creatine kinase. Statistical analysis was performed using two-way ANOVA followed by Tukey's post hoc test in (C), Fisher's exact test in (E), and Student's *t*-test in (F). NS: No significance.

A previous study raised concerns about fatal arrhythmia caused by the pro-regenerative microRNA therapy via serotype 6 adeno-associated virus-mediated gene delivery in the ischemic myocardium of swine 15. To investigate the safety of the miR-125a-5p agomir, we subcutaneously implanted an insertable cardiac monitor (ICM) and monitored the cardiac electrical activity of the myocardial I/R swine throughout the 28-day experimental period (**Figure 8A**). One swine in the I/R control, NC agomir, and miR-125a-5p groups died at an early stage (day 2-3) because of myocardial I/R injury-induced fatal sustained ventricular tachycardia (**Figure 8B**). No significant difference in the average heart rate of the NC agomir and the miR-125a-5p swine was detected at baseline, day 3, and day 28 post-myocardial I/R (**Figure 8C**). Importantly, electrocardiogram (ECG) analysis demonstrated several types of arrhythmia episodes during the observation period, including paroxysmal supraventricular tachycardia (ST), atrial tachycardia (AT), atrial fibrillation (AF), and paroxysmal ventricular tachycardia (VT), but no significant difference in the number of arrhythmia events was observed between the NC agomir and the miR-125a-5p groups (**Figure 8D** and** 8E**). Additionally, there was no significant change observed in blood chemistry indicators (**Figure 8F**). Taken together, these data demonstrate that the delivery of miR-125a-5p agomir in porcine I/R hearts is both feasible and safe, and does not increase the burden of arrhythmia or hepatic, renal, or cardiac toxicity.

## Discussion

Ischemic heart diseases produce immense health and economic burdens globally 29, and multiple therapeutic attempts have been made to overcome this dilemma 30, 31. Here, this study reveals that (i) the cardioprotective role of MSCs and MSC-Exos against I/R injury can be reproduced by the agomir of their contained active ingredient miR-125a-5p to a certain extent in mice; (ii) miR-125a-5p agomir attenuates cardiac remodeling and improves myocardial function, which is associated with the increase in M2 macrophage polarization, promotion of angiogenesis, and inhibition of fibroblast proliferation and activation; (iii) the mechanisms by which miR-125a-5p agomir modulates the functions of macrophages, fibroblasts, and ECs are related to the inhibition of KLF13 expression and ERK1/2 pathway, TGFBR1 expression, and DAAM1 expression, respectively; and (iv) the administration of miR-125a-5p agomir in myocardial I/R swine improves cardiac performance and remodeling, with no increases in the frequency of arrhythmia or toxicity in the liver, kidney, or heart. These findings provide evidence that treatment with miR-125a-5p agomir is a safe and effective option for stem cell- and exosome-based therapies in the I/R myocardium.

The efficacy of MSC therapy in clinical heart failure has been demonstrated by a previous study, which attributed the therapeutic role of MSCs to their engrafting and differentiation ability 32. Indeed, only a few MSCs survive for more than 1 week after systemic treatment, indicating that paracrine mechanisms are key to the function of MSCs 33. Recently, MSC-Exo therapy has received attention in cardiac repair in the ischemic myocardium and compensates for the deficiency of MSCs 11, 30. However, the content of MSC-Exos varies greatly from batch to batch, and the complex content may bring unintended side effects. For instance, uncontrolled exchange of genetic information between cell populations via exosomes has been reported 34. Furthermore, exosomes also induce an immune response as the presentation of key antigens 35. As the main agent involved in MSC-Exo function, microRNA targets a single or few genes, and it is unlikely for a single-component microRNA to induce alterations in host genetic information and immune response. Our *in vitro* experiments show that the agomir of MSC-Exo-enriched miR-125a-5p has better transfection efficiency than miR-125a-5p mimics, and that treatment with miR-125a-5p agomir in a mouse model with myocardial I/R exerts a cardioprotective effect that rivals the benefit of MSC or MSC-Exo delivery, including improvements in survival, myocardial function, and cardiac remodeling, indicating that intramyocardial delivery of miR-125a-5p agomir is an effective therapeutic strategy for ischemic heart disease.

Although we confirmed that miR-125a-5p, MSC-Exos, and MSCs all have cardioprotective abilities against I/R injury, this study was not designed to directly compare the biological efficacy of the three treatments, as none of them were performed under identical or optimal conditions. For example, immunosuppression, which is required for the experimental use of MSCs and MSC-Exos as allogeneic cell and component transplants, and which we did not use in this study, would affect the efficacy of MSCs and MSC-Exos, but not that of miR-125a-5p agomir. Furthermore, we did not accurately confirm the optimal dose of these three treatments; therefore, we only sought to demonstrate that miR-125a-5p agomir has a protective effect and may provide a therapeutic option for myocardial I/R injury such as that provided by MSC and MSC-Exo treatments, and not to compare which treatment is more effective. MSC-Exos contain several microRNA components that exert myocardial protective effects. The aim of this study was to propose a novel idea to address the shortcomings of clinically relevant cell and exosome therapies, such as low cell transplantation rates, unstable exosome content, and immune rejection, to screen for an active exosome component, and ultimately to develop a simple and effective single microRNA drug for the treatment of ischemic heart disease. As several clinical studies have reported on the use of MSCs for the treatment of MI or I/R 32, 36, the most abundant miR-125a-5p in MSC-Exos was selected for oligonucleotide synthesis in this study.

It is widely recognized that there exist two typical subpopulations of macrophages: classically activated M1 macrophages and alternatively activated M2 macrophages 37. M2 macrophages are involved in myocardial wound repair post-cardiac I/R (lasting days to weeks) 9, and the strategy of polarizing macrophages toward a reparative M2 phenotype is instrumental in long-term infarction healing 38. Importantly, previous studies have indicated the beneficial effects of early initiation of anti-inflammatory therapy in the treatment of urgent MI requiring revascularization, suggesting the need for an early inflammatory suppression strategy in myocardial I/R 39. Therefore, we propose that early inflammatory intervention should be performed in the I/R myocardium by promoting the polarization of M2 macrophages. In addition to previous studies reporting that miR-125a-5p modulates the polarization of macrophage colony stimulating factor-induced bone marrow-derived macrophages 12 and participates in the regulation of macrophage function in inflammatory diseases and diabetic retinopathy 40, we further prove that miR-125a-5p agomir delivery regulates macrophage polarization not only *in vitro*, but also in myocardial I/R mice and large animals. FCM and immunostaining analyses also demonstrated that miR-125a-5p agomir significantly promotes M2 macrophage polarization and inhibits M1 macrophage polarization, ultimately increasing the M2/M1 ratio.

KLF13 is a cardiac transcription factor that is involved in heart development. The inhibition of KLF13 expression in adult hearts has been reported to regulate the polarization of stimulated macrophages 41. The nuclear translocation of KLF13 has been reported to be involved in T lymphocyte activation by upregulating CC chemokine ligand-5 (CCL5) expression 42, which can directly activate M1 macrophage polarization and impede M2 polarization via CCR1- and CCR5-mediated activation of MAPK pathways 43. Consistently, our results identify *Klf13* as one of the target genes of miR-125a-5p to promote M2 macrophage polarization, and miR-125a-5p agomir treatment significantly decreases the expression and nuclear translocation of KLF13 *in vitro* and *in vivo*. Lentiviral KLF13 overexpression reverses the promotion of M2 marker expression and partially abolishes the improved cardiac function induced by miR-125a-5p agomir, indicating that the effects of miR-125a-5p on macrophage polarization and subsequent myocardial protection may be mediated, at least partially, by KLF13. Akt and MAPK family kinases are commonly implicated in the stress-activated signaling pathways and are believed to be of great importance in cardiac ischemic preconditioning and cardiac remodeling in the I/R myocardium 24, 25. Among them, ERK1/2 inactivation has been reported to be associated with the suppression of macrophage polarization toward the M1 phenotype induced by celastrol, a plant-derived triterpene 44. In addition, the cardioprotective effects of ethanol treatment in hearts subjected to I/R are associated with the inhibition of ERK1/2 activity 45. In this study, we determined that the inactivation of ERK1/2 but not the other three kinases is in response to the treatment with miR-125a-5p agomir, and that pharmacological activation of ERK1/2 can reverse the regulation of macrophage polarization by miR-125a-5p. Therefore, the regulation of ERK1/2 may be another mechanism of miR-125a-5p-mediated macrophage polarization in this myocardial I/R model.

It has recently been discovered that fibroblasts are activated post-MI or I/R to secrete cytokines and chemokines, as well as extracellular matrix components, that form infarct scars and promote cardiac fibrosis 46. Excessive activation of fibroblasts can result in increased LV wall stiffness and reduced mechano-electric coupling to adversely induce LV remodeling and impair cardiac function. Therefore, prompt inhibition of fibroblast activation is considered crucial for the remission of cardiac remodeling after MI or I/R 47, 48. Our results demonstrate that miR-125a-5p agomir decreases the proliferation and activation of cardiac fibroblasts and alleviates cardiac fibrosis by inhibiting the expression of TFGBR1, which blocks the activation of the TGF-β signaling pathway in fibroblasts. Moreover, TGFBR1 is reported to be a prognostic biomarker after acute MI, and its expression is significantly elevated post-MI and shows a remarkable positive correlation with the extent of cardiac remodeling 49. In the present study, we also found that miR-125a-5p promotes *in vitro* injured EC function and *in vivo* angiogenesis of the I/R myocardium by targeting *Daam1* and repressing the expression of its encoded DAAM1 protein. Previous studies have reported that DAAM1 induces actin polymerization and microtubule stabilization of ECs, thereby inhibiting EC proliferation, migration, and angiogenesis 50. Thus, miR-125a-5p-mediated angiogenesis may be associated with actin and microtubule dissociation induced by DAAM1 inhibition. Hence, the reduction in fibroblast proliferation and activation via suppression of TGFBR1 and the promotion of angiogenesis by inhibiting DAAM1 have also been identified as important mechanisms underlying the protection of the I/R myocardium by miR-125a-5p.

As miR-125a-5p has no significant effect on cardiomyocyte apoptosis *in vitro*, we infer that the *in vivo* reduction in cardiomyocyte apoptosis observed in the miR-125a-5p agomir-injected I/R myocardium is likely to be secondary. A direct evidence is that macrophage depletion by Cl_2_MDP-Lipo abolishes part of the miR-125a-5p agomir-induced improvements in cardiomyocyte apoptosis in the I/R heart. Additionally, it has been reported that the inflammatory suppression of M2 macrophages in the I/R myocardium is associated with cell repair activation, including the inhibition of cardiomyocyte apoptosis 9. Furthermore, the abrogation of TGF-β signaling is associated with not only the reduction of fibroblast proliferation and activation but also the alleviation of cardiomyocyte hypertrophy and apoptosis 51. The promotion of cardiac angiogenesis is also instrumental in intervening in cardiomyocyte apoptosis 52. In summary, the effects of miR-125a-5p on inflammatory responses and cardiomyocyte apoptosis observed in our experiments may be mediated, at least in part, by the regulation of macrophage polarization, fibroblast proliferation and activation, and endothelial cell function.

Ventricular arrhythmias are a major contributor to cardiovascular mortality in patients with myocardial I/R, and the risk of arrhythmia is the most concerning safety issue when developing a novel treatment strategy. Multiple studies have reported that the transplantation of induced pluripotent stem cell or embryonic stem cell-derived cardiomyocytes into non-human primate hearts increases the occurrence of ventricular tachycardia 53, 54. Consistently, uncontrolled delivery of miR-199a promotes cardiomyocyte proliferation in myocardial I/R swine, but also increases the risk of ventricular fibrillation 15. This study showed that the swine died of ventricular tachycardia in both the miR-125a-5p group and the control group, while no significant difference between the two groups was observed, indicating that death was caused by the injury of the I/R myocardium itself. Additionally, we found no significant difference in the incidence of other arrhythmia episodes or liver, kidney, or cardiac toxicity between the two groups. These results demonstrate that the application of miR-125a-5p agomir in porcine I/R myocardium is safe.

MicroRNA agomir is a well-established microRNA delivery tool whose chemical modifications endow it with better tissue permeability and microRNA stability *in vivo*. In this study, we used intramyocardial injection for microRNA delivery, which ensured accurate delivery and high local expression of microRNA in the border zone. However, considering that intramyocardial injection is not convenient in clinical treatment, how to improve the targeted efficacy and operational availability of microRNAs delivery in MI or the I/R myocardium requires further investigation. Meanwhile, some reports are incompatible with our findings regarding the functions of miR-125a-5p 55-58. These inconsistencies may be caused by different disease models, animal species, and experimental conditions.

## Conclusions

Taken together, this study provides a proof of concept that the application of miR-125a-5p agomir is an effective and safe strategy, and that this microRNA therapy with a single component, a definite mechanism, and no immunogenicity may serve as a promising approach for cardiac repair after I/R.

## Supplementary Material

Supplementary methods, figures and tables.
