# Supplementary material for: Therapeutic delivery of microRNA-125a-5p oligonucleotides improves recovery from myocardial ischemia/reperfusion injury in mice and swine
Source: Theranostics. 2023 Jan 1;13(2):685–703. doi: 10.7150/thno.73568 (PMC9830430; doi:10.7150/thno.73568)
Supplement: Supplementary file 1 — Supplementary methods, figures and tables. [file thnov13p0685s1.pdf]

Supplementary Materials and Methods

Mesenchymal Stem Cell (MSC) Isolation, Culture, and Characterization

Mouse bone marrow-derived MSCs were isolated, cultured, and verified as described previously [1]. Briefly, bone marrow was obtained by repeated flushing of the tibia and femoral cavity, and the aspirate was then centrifuged at 400 relative centrifugal force (rcf) for 30 min at 20°C. After washing twice with PBS, the cells were resuspended in alpha minimal essential medium (α-MEM) (Thermo Fisher Scientific, 32571036) supplemented with 10% fetal bovine serum (FBS) (Thermo Fisher Scientific, 10100147), plated on a 100-mm culture dish, and placed in a CO_2 incubator (Thermo Fisher Scientific) containing 5% CO_2 and 21% O_2 at 37°C. MSCs were used for follow-up experiments after five passages.

For MSC verification, cells were trypsinized and washed with phosphate buffered saline (PBS) (Thermo Fisher Scientific, 10010072). Antibodies (CD73, CD90, CD105, CD14, CD34, CD45, and human leukocyte antigens class II-DR [HLA-DR]) were co-incubated with 1×10^6 MSCs for 30 min at 4°C. The antibodies used in this study are listed in **Table S1**. After resuspension in 200 μL staining buffer (BD Biosciences, 554656), MSCs were analyzed by a CytoFLEX flow cytometer (Beckman) and FlowJo software (v10.4, BD Biosciences) to investigate the surface molecular markers. Osteogenic differentiation was conducted using α-MEM supplemented with 10% FBS, 0.1 μM dexamethasone, 10 mM β-glycerophosphate, and 50 μM ascorbic acid 2-phosphate. For chondrogenic differentiation, the culture medium was replaced with Dulbecco's Modified Eagle Medium (DMEM) high glucose (Thermo Fisher Scientific, 11965118) supplemented with 10% FBS, 1 × Insulin-Transferrin-Selenium, 0.1 mg/mL sodium pyruvate, 0.04 mg/mL L-proline, 0.05 mg/mL ascorbic acid-2 phosphate, 0.1 nM dexamethasone, and 0.1 mg/mL transforming growth factor-beta (TGF-β). For adipogenic differentiation, the medium was replaced with DMEM high glucose supplemented with 10% FBS, 0.1 μM dexamethasone, 0.2 mM indomethacin, 0.01 mg/mL insulin, and 0.5 mM 3-isobutyl-1-methylxanthine. All reagents were purchased from Sigma-Aldrich. On day 21, osteogenic, chondrogenic, and adipogenic differentiation were further determined by Alizarin Red (Sigma-Aldrich, A5533), Alcian Blue (Sigma-Aldrich, A5268), and Oil Red O (Sigma-Aldrich, O0625) staining, respectively, according to the manufacturer's instructions.

MSC-derived Exosome (MSC-Exo) Isolation and Characterization

Exosomes used for animal and *in vitro* experiments were collected and concentrated from MSCs through differential ultracentrifugation and verified as described previously [2]. Briefly, MSCs were cultured to 70%–80% confluency, at which point, the culture medium was replaced with α-MEM supplemented with 10% exosome-depleted FBS (Thermo Fisher Scientific, A2720801) and cultured for 48 h.

The medium was then collected and centrifuged at 2,000 rcf for 10 min and at 10,000 rcf for 30 min. After passing through a 0.22-μm filter (Millipore, SLGP033RB) to remove the cell debris, the supernatant was centrifuged at 100,000 rcf for 70 min, and the pellet was resuspended in PBS. The mixture was centrifuged again at 100,000 rcf for 70 min, and the pellet containing isolated exosomes was resuspended in PBS and stored at –80°C for further use. The exosome concentration was determined by BCA assay (Beyotime Biotech, P0012). Exosomes from cardiac fibroblasts were collected as a control.

For MSC-Exo characterization, exosomes were dissolved in 1 mL PBS, and the particle concentration and size distribution were evaluated via a NanoSight LM10 apparatus (Malvern). The expression of exosomal marker proteins (CD63, CD9, TSG101, and Alix) was determined by immunoblotting. The exosomal ultrastructure was assessed via transmission electron microscopy (TEM) as described previously [3]. Briefly, the resuspended exosomes were fixed with 2.5% glutaraldehyde, post-fixed in buffered 1% OsO_4 with 1.5% $K_4Fe(CN)_6$, embedded in 1% agar, and processed according to the standard Epon812 embedding procedure. Exosomes were visualized on thin sections (60 nm) via a Tecnai G2 T12 transmission electron microscope (FEI).

Next Generation Sequencing (NGS)

microRNA sequencing and NGS data analysis of exosome samples were performed by LC Sciences [4]. The total exosomal RNA was purified via a miRNeasy kit (QIAGEN, 217684) according to the manufacturer's protocols, and the quality and quantity of RNA were analyzed by a 2100 Bioanalyzer (Agilent). Sequencing libraries were generated through the TruSeq Small RNA Sample Preparation protocol, which includes the ligation of specific RNA adapters to both the 3′ and 5′ ends for each sample, followed by reverse transcription, amplification, and purification of small-RNA libraries (size range 22 to 30 nucleotides). Sequencing was performed on an Illumina HiSeq 2500 platform, and adapter dimers, junk, low complexity, common RNA families (rRNA, tRNA, snRNA, snoRNA), and repeats were removed from the raw reads with an in-house program, ACGT101-miR (LC Sciences). Unique sequences with lengths of 18 to 26 nucleotides were mapped to specific species precursors in miRBase 22.0 by BLAST search to identify known microRNAs and novel 3p- and 5p- derived microRNAs; length variations at both the 3′ and 5′ ends and one internal sequence mismatch were allowed in the alignment. The unique sequences mapping to specific species of mature microRNAs in hairpin arms were identified as known microRNAs. Unique sequences mapping to the other arm of known specific species precursor hairpin opposite to the annotated mature microRNA-containing arm were deemed novel 3p- or 5p-derived microRNA candidates. The remaining sequences were mapped to other selected species precursors (except specific species)

in miRBase 22.0 by BLAST search, and the mapped pre-microRNAs were further BLASTed against the specific species genomes to determine their genomic locations. Unmapped sequences were BLASTed against the specific genomes, and the hairpin RNA structures containing sequences were predicated from the flank 80 nucleotide sequences using RNAfold software (http://rna.tbi.univie.ac.at/cgi-bin/RNAWebSuite/RNAfold.cgi). The relative expression of the 14 upregulated miRNAs and 5 downregulated microRNAs in MSC-Exos compared to FB-Exos were displayed in a heat-map, and data were expressed as fold change (log_2) of exosomal microRNAs normalized read counts (**Table S2**). The microRNA sequencing data have been deposited in the Genome Sequence Archive in the National Genomics Data Center (Nucleic Acids Res 2022), China National Center for Bioinformation/Beijing Institute of Genomics, Chinese Academy of Sciences (project number: PRJCA007694, GSA accession number: CRA005702), and are publicly accessible at https://ngdc.cncb.ac.cn/gsa.

MiR-125a-5p Inhibition in MSCs and MSC-Exos

Synthetic mmu-miR-125a-5p antagomir or negative control (NC) antagomir was mixed with culture medium to form a final antagomir concentration of 200 nM. Next, antagomir was transfected directly into MSCs for 24 h, and cells and exosomes depleted with miR-125a-5p were isolated for further experiments.

Animals

All animal procedures and protocols (mice and swine) were performed in accordance with the Guide for the Care and Use of Laboratory Animals published by the U.S. National Institutes of Health (8th Edition, 2011) and approved by the Institutional Animal Care and Use Committee of Tongji University. Female C57BL/6 mice (20 g, 6–8 weeks old) were obtained from Shanghai Jiesijie Laboratory Animal Co., Ltd, and Bama swine (female, 20 kg, 16–20 weeks old) were purchased from Shanghai Jiagan Laboratory Animal Co., Ltd. The animals were fed a standard laboratory diet and maintained in a 12-h light/12-h dark cycle.

Myocardial Ischemia/Reperfusion (I/R) Surgery and Treatment in Mice

The mouse model with myocardial I/R was established surgically by ligation of the left anterior descending (LAD) coronary artery as previously described [5]. Briefly, mice were placed in an induction chamber and then connected to a small rodent ventilator with 2% isoflurane. Subsequently, the chest was opened to expose the heart, left auricle, and LAD. The LAD was ligated with an 8-0 silk suture approximately 2 mm below the edge of the left auricle for 60 min, followed by reperfusion. Ligation was considered successful when the anterior wall of the left ventricle (LV) turned pale. The experimental treatments were administered at the onset of reperfusion. Animals

in groups with different doses of miR-125a-5p agomir and MSC were intramyocardially injected with a concentration gradient of miR-125a-5p agomir (10, 20, and 40 nmol) or MSC (1×10^5, 3×10^5, and 5×10^5) in 15 μL PBS (similarly hereafter), respectively; animals in the I/R control, MSC, and MSC-Exo groups were injected with 15 μL PBS, 5×10^5 MSCs, and 10 μg MSC-Exos, respectively; animals in the miR-125a-5p and NC agomir groups were administered with 20 nmol (~8 mg/kg of 20 g) mmu-miR-125a-5p agomir, and 20 nmol NC agomir, respectively; animals in the $MSC^{125a\text{-}anta}$ and $MSC^{NC\text{-}anta}$ groups were treated with 5×10^5 MSCs transfected with mmu-miR-125a-5p antagomir and NC antagomir, respectively; and animals in the $MSC^{125a\text{-}anta}$-Exo and $MSC^{NC\text{-}anta}$-Exo groups were treated with 10 μg exosomes isolated from $MSC^{125a\text{-}anta}$ and $MSC^{NC\text{-}anta}$, respectively. Next, the thoracotomy site was closed in layers with 6-0 sutures. After reinstallation of spontaneous respiration, the mice were extubated and allowed to recover from anesthesia. Animals were maintained on a 37°C heating pad during the myocardial I/R surgery until recovery. All agents were directly injected into the border zone of the I/R hearts at the onset of reperfusion, and sham-operated mice underwent the same procedure, excluding LAD ligation. At the end of the experiment, in addition to the hearts used for flow cytometry (FCM) analysis, the remaining hearts were cut in half; half was used for histological studies, and the other half was used for RNA and protein extraction and enzyme-linked immunosorbent assay (ELISA).

To deplete the macrophages in the myocardium, mice were intraperitoneally injected with 200 μL Cl_2MDP-Lipo (Liposoma, C-005) 1 day prior to and 1 day following myocardial I/R surgery. PBS-Lipo (Liposoma, P-005) was used as a control. For specific KLF13, TGFBR1, or dishevelled-associated activator of morphogenesis 1 (DAAM1) overexpression in the heart, KLF13, TGFBR1, or DAAM1 overexpression (KLF13-OE, TGFBR1-OE, or DAAM1-OE) or negative control (KLF13-NC, TGFBR1-NC, or DAAM1-NC) plasmid was successfully constructed and then packaged into HEK 293T cells by GeneChem. Then, lentiviruses (1×10^9 TU/mL) in 15 μL enhanced infection solution (GeneChem) were delivered into the mouse myocardium 7 days prior to myocardial I/R surgery and agomir treatment. For extracellular-regulated kinase 1/2 (ERK1/2) activation, LY2828360 (MedChemExpress, HY-16642A) was injected intraperitoneally into mice (3 mg/kg/day × 12 days) before the myocardial I/R surgery [6]. Dimethyl sulfoxide (DMSO) was used as the vector.

Echocardiography

The cardiac function of mice was verified via echocardiography with a Vevo 2100 system (FUJIFILM Visual-Sonics). Two-dimensional-guided M-mode images were used to determine the LV internal diameter at end-systole (LVIDs), the LV internal

diameter at end-diastole (LVIDd), the LV end-diastolic volume (LVEDV), and the LV end-systolic volume (LVESV). The LV ejection fraction (LVEF) was derived as LVEF (%) = (LVEDV – LVESV)/LVEDV × 100%. The LV fractional shortening (LVFS) was calculated as LVFS (%) = (LVIDd – LVIDs)/LVIDd × 100%. All measurements were based on the average of at least five independent cardiac cycles.

FCM Analysis

Non-cardiomyocytes were collected from mouse hearts as described previously [7]. Mononuclear cells, including macrophages, were then isolated from non-cardiomyocytes using a centrifugal-based method (500 rcf for 30 min) with percoll gradients (Cytiva, 17089102) [8]. Cells were blocked with mouse Fc block (BD Biosciences, 553141) for 5 min at 4°C, incubated with primary and isotype control antibodies for 30 min at 4°C, resuspended in 200 μL staining buffer, and evaluated using a CytoFLEX flow cytometer and FlowJo software.

Cell Culture and Treatment

The murine macrophage RAW 264.7 cell line was purchased from ATCC (TIB-71). Primary neonatal mouse cardiomyocytes were isolated from 1- to 2-day-old C57BL/6 mice as previously reported [9]. Cardiac fibroblasts and endothelial cells (ECs) were isolated from 6- to 8-week-old C57BL/6 mice via a published method [7]. RAW 264.7 macrophages were stimulated with 100 ng/mL lipopolysaccharide (LPS) for 6 h, followed by treatment with 100 nM Cy3 dye-labeled miR-125a-5p agomir, 100 nM miR-125a-5p agomir, 200 nM miR-125a-5p antagomir, or corresponding NC for 24 h before further analyses. Primary neonatal mouse cardiomyocytes were treated with Cy3-miR-125a-5p agomir, miR-125a-5p agomir, miR-125a-5p antagomir, or the corresponding NC, and then some were subjected to 6 h of oxygen-glucose deprivation (OGD; glucose-free DMEM plus hypoxic atmosphere) followed by 18 h of recovery (OGD/R). ECs were also treated with Cy3-miR-125a-5p agomir, miR-125a-5p agomir, miR-125a-5p antagomir, or the corresponding NC, and some were subjected to a hypoxic atmosphere (1% O_2) for 24 h. Calcein (Beyotime Biotech, C2012) (2 μg/mL) was used to assess the tube formation of ECs. For DAAM1 overexpression in ECs, DAAM1-OE lentiviruses (1×10^8 TU/mL) were co-cultured with ECs for 24 h, and DAAM1-NC lentiviruses served as a control. TGF-β1 (20 ng/mL; R&D Systems, 240-GMP)-stimulated or non-TGF-β1-stimulated cardiac fibroblasts were treated with Cy3-miR-125a-5p agomir, miR-125a-5p agomir, miR-125a-5p antagomir, or the corresponding NC for 24 h. To overexpress TGFBR1 in cardiac fibroblasts, cells were co-cultured with TGFBR1-OE lentiviruses (1×10^8 TU/mL) for 24 h before replacement with normal medium, and TGFBR1-NC lentiviruses were used as a control.

Luciferase Reporter Assay

The *Klf13* sequences containing 233 to 239 bp, *Tgfbr1* sequences containing 2243 to 2249 bp, and *Daam1* sequences containing 1146 to 1153 bp were amplified and cloned between the *XhoI* and *SalI* sites of the pmirGLO dual-luciferase microRNA target expression vector (Promega, E1330). The mutant *Klf13*, *Tgfbr1,* or *Daam1* 3′UTR pmirGLO vector was generated using a QuikChange II XL Site-Directed Mutagenesis Kit (Stratagene, 200522) according to the manufacturer's instructions. RAW 264.7 cells, cardiac fibroblasts, and ECs were cultured until 60% confluency and then co-transfected with miR-125a-5p agomir and either the *Klf13*, *Tgfbr1*, or *Daam1* 3′UTR pmirGLO vector or the mutant *Klf13*, *Tgfbr1* or *Daam1* 3′UTR pmirGLO vector, respectively. Cells transfected with the no-insert pmirGLO vector were used as a control. Twenty-four hours after transfection, the cells were analyzed in a luciferase activity using a Dual-Glo Luciferase Assay System (Promega, E2920). The relative luciferase activity was determined by the ratio of firefly luminescence/Renilla luminescence.

Myocardial I/R Surgery and Treatment in Swine

Porcine myocardial I/R surgery was performed as previously described [4]. Briefly, animals were anesthetized with inhaled 2% isoflurane, intubated, and ventilated with a respirator and supplemental oxygen. Body temperature, electrocardiogram (ECG), blood pressure, and arterial oxygen saturation were monitored throughout the surgical procedure. After left thoracotomy, the pericardial sac was opened to expose the LAD and its diagonal branches, and the LAD was occluded for 60 min at a site between the 1st and 2nd diagonal branches and then reperfused. Ligation was considered successful when the anterior wall of the LV turned pale and ST elevation was observed on ECG. The experimental treatments were administered at the onset of reperfusion. Animals in the miR-125a-5p group received 1.2 μmol (~0.5 mg/kg of 20 kg) hsa-miR-125a-5p agomir (in 1 mL PBS, similarly hereafter); NC agomir animals received 1.2 μmol NC agomir; animals in the I/R control group received 1 mL PBS; and animals in the sham group underwent all surgical procedures except occlusion and recovered without any experimental treatment. After closing the chest in layers, a Reveal LINQ insertable cardiac monitor (ICM) device (Medtronic) was implanted subcutaneously to monitor the electrical activity. Animals in all treatment groups received standard postoperative care, including analgesia and antibiotic administrations, until the animals ate normally and became active.

Telemetric Monitoring

The electrical activity from conscious swine was continuously monitored by a Reveal LINQ ICM device (Medtronic), which was implanted subcutaneously after the surgery and treatment. The arrhythmic events and heart rates were recorded. A tachy

episode was defined as a rate faster than 100 beats/min; heart pause was defined as a complete disappearance of electrical activity; a brady episode was defined as a rate slower than 60 beats/min; paroxysmal supraventricular tachycardia (ST) was defined as narrow QRS complexes (< 120 ms) with regular tachycardia rhythm (160–220 beats/min), and hidden or inverted P waves [10, 11]; atrial tachycardia (AT) and atrial fibrillation (AF) were automatically recognized by the ICM device; paroxysmal ventricular tachycardia (VT) was defined as ≥ 3 consecutive ventricular beats that occurred at a rate faster than 100 beats/min, or continuous ventricular beats that terminated spontaneously within 30 s; ventricular fibrillation (VF) was defined as a chaotic tachycardia without consistently identifiable QRS complexes; and sustained ventricular tachycardia was defined as sustained ventricular beats lasting longer than 30 s [12].

Cardiac Multi-Detector Computed Tomography (MDCT)

MDCT images were obtained 28 days after myocardial I/R surgery and treatments. Animals were anesthetized with 2% isoflurane, positioned in a supine position, and scanned with ECG monitoring by an Aquilion ONE TSX-301C MDCT scanner (Canon Medical). A 35-mL bolus of Iopamidol (Bracco Sine, Iopamiro 370) was injected intravenously opacifiying the LV chamber at a rate of 3.5 mL/s during the first phase acquisitions. An additional 25 mL of Iopamidol was subsequently administered at a rate of 1.0 mL/s, and delayed enhancement (de)-MDCT images were acquired with a coronary CT angiography model 7 min after contrast delivery (second phase acquisitions). Images were acquired using the following parameters: gantry rotation time = 275 ms, detector collimation = 0.5 mm × 320 (isotropic voxels = 0.5 × 0.5 × 0.5 mm^3), tube voltage = 100 kV, and tube current = 400 mA.

All raw data were reconstructed in 5% steps from 0%–95% throughout the R-R interval, and images in optimal phase were reformatted separately for myocardial perfusion (first-pass images) and scar assessment (de-MDCT images). Cardiac functional parameters, including LVEF, stroke volume, LVESV, LVEDV, wall motion, and myocardial perfusion, were evaluated on a Vitrea 2 computer workstation (Vital Images). The infarct size on day 28 post-cardiac I/R was determined by six cross-sections of the de-MDCT images from the apex to base by dividing the infarct area by the total LV area.

Hemodynamic Measurements

Invasive monitoring of aortic and LV pressure, and LV function of the anesthetized animals was acquired via a transvascular (through the left external carotid artery) polyvinyl chloride catheter introduced into the LV lumen as previously described [4]. Aortic and LV pressures were continuously monitored with a PowerLab system (AD

Instrument), and LV end-diastolic pressure (LVEDP), peak contraction velocity (dP/dt_{max}), and peak relaxation velocity (dP/dt_{min}) measurements were made on day 28 after myocardial I/R. After the measurement, blood samples were drawn and blood tests were performed by the Department of Laboratory Medicine, Shanghai East Hospital, Tongji University School of Medicine.

RNA Isolation and Quantitative Reverse Transcription Polymerase Chain Reaction (RT-qPCR)

Total RNA and microRNA were extracted by TRIzol reagent (Thermo Fisher Scientific, 15596018) following the manufacturer's protocols. The concentrations of RNA were determined using a NanoDrop 2000 spectrophotometer (Thermo Fisher Scientific), and samples with an A260/A280 ratio from 1.8 to 2.0 were applied for reverse transcription. mRNAs were reverse transcribed using a PrimeScript RT Reagent Kit with gDNA Eraser (TaKaRa, RR047A). Then, 20-μL reactions, including 1 μg mRNA and primers, were detected by a QuantStudio 7 Flex system (ABI) using PowerTrack SYBR Green Master Mix (ABI, A46109). microRNAs were reverse transcribed with a TaqMan microRNA Reverse Transcription Kit (ABI, 4366596) following a quantitative analysis of microRNAs by TaqMan Advanced microRNA Assays (ABI, 4440887). The PCR thermocycling conditions were 95°C for 1 min, followed by 40 cycles of 95°C for 10 s and 60°C for 32 s. The relative expression of microRNAs was normalized to *U6* or cel-miR-39, and the relative mRNA expression was normalized to *Gapdh* and calculated using the standard $2^{-(\Delta Ctsample-\Delta Ctcontrol)}$ method. The primers used for PCR are listed in **Table S3**.

Histological Studies

2,3,5-triphenyltetrazolium Chloride (TTC) Staining

Mouse hearts were collected 3 days after I/R injury and cut into five slices perpendicular to the LAD from the apex to base, and slices were immediately immersed in 1% TTC (Sigma-Aldrich, T8877) at 37°C in the dark for 10 min to distinguish infarcted tissue from viable myocardium.

Tissue Sample Preparation

Mouse heart tissues were harvested and perfused with PBS, followed by embedding with Tissue-Tek O.C.T. Compound (SAKURA, 4583) on liquid nitrogen, and subsequently subjected to serial cryosectioning (8 μm-thickness). For porcine hearts, the LV wall was cut vertically into six rings (R1 to R6) from the apex to base, and each ring was cut into eight samples (S1 to S8) in sequence. Then, the tissues from S1 to S5 of R2 to R4 (i.e., from the site of agomir injection) were embedded and cryosectioned.

Masson’s Trichrome Staining

Tissue sections were stained using a Masson’s trichrome Stain Kit (Solarbio, G1340) according to the manufacturer’s instructions and observed under an optical microscope. For mouse tissues, histological evaluation of infarct size (at day 3 post-myocardial I/R) and fibrosis size (at day 28) were calculated as a percentage of the infarct area (or fibrosis area)/total LV area. The severity of fibrosis was classified as follows: > 45% fibrotic area, ‘‘severe;’’ 25%–45% as ‘‘moderate;’’ or < 20% as ‘‘mild’’ for I/R hearts [13]. For porcine tissues, interstitial fibrosis was determined by the ratio of fibrosis area/each random high-power field of view.

Hematoxylin and Eosin (H&E) Staining

Tissue sections were stained using a H&E Staining Kit (Beyotime Biotech, C0105) following the manufacturer’s protocols before being observed under an optical microscope. The infiltration of inflammatory cells was evaluated by the ratio of inflammatory cell area (white dotted)/each random high-power field of view.

Immunostaining

Heart sections or cell slides were fixed with 4% paraformaldehyde, permeabilized in 0.25% triton X-100, blocked in Ultra-V Block buffer for 10 min at room temperature, incubated overnight at 4°C with primary antibodies, and then incubated for 2 h at room temperature with the corresponding secondary antibodies. For wheat germ agglutinin (WGA) staining, sections were stained for 1 h at room temperature using a FITC-labeled WGA dye (Sigma-Aldrich, L4895). For terminal deoxynucleotidyl transferase dUTP nick end labeling (TUNEL) staining, sections were stained with an In Situ Cell Death Detection Kit (Roche, 12156792910) according to the manufacturer’s instructions. Nuclei were counterstained with DAPI (Beyotime Biotech, P0131), and images were taken using a TCS SP8 STED 3X confocal microscope (Leica). To analyze the capillary density, sections were immunofluorescently stained for CD31 and cardiac troponin I (cTnI), and images were analyzed by ImageJ software (v1.8.0, NIH) to determine the number of capillaries surrounding each cardiomyocyte. The positively stained cells or areas were counted in 3 to 5 sections per heart, and 4–10 high-power fields per section.

ELISA

Three days and 7 days (in mice) after the myocardial I/R procedure, the heart tissue and whole blood samples from mice and swine were collected. Blood samples were centrifuged at 1,000 rcf for 10 min to obtain serum, and the heart tissue was homogenized using an electric homogenizer. The protein levels of IL-6 and IL-10 in mouse serum and heart tissue were determined using IL-6 (Biolegend, 431315) and IL-10 (Biolegend, 431425) ELISA kits. The concentrations of IL-6 and IL-10 in

swine were detected by IL-6 (Abcam, ab100755) and IL-10 (Bertin Bioreagent, A05414) ELISA kits, followed by the manufacturer's protocols.

Western Blotting

Heart tissue proteins were isolated using an electric homogenizer and RIPA lysis buffer (Beyotime, P0013) supplemented with 1 mM phenylmethylsulfonyl fluoride (PMSF) (Beyotime, ST506). Protein extracts (20 µg) in each lane were separated by gel electrophoresis, transferred to a polyvinylidene fluoride membrane (Millipore, IPVH00010), blocked by 5% skimmed milk for 2 h, and then incubated with primary antibodies overnight at 4°C. The membranes were incubated with HRP-conjugated secondary antibodies for 2 h at room temperature and exposed through enhanced chemiluminescence.

Statistical Analysis

Data are presented as the mean ± standard error of mean (SEM). Quantitative analysis of experimental images was performed using ImageJ software. Differences between two mean values were evaluated by Student's *t*-test, and multiple comparisons at a single time point were determined by one-way analysis of variance (ANOVA) followed by Tukey's post hoc test. To evaluate differences between groups at multiple time points, data were analyzed using two-way ANOVA followed by Tukey's post hoc test. For survival analysis, a Kaplan–Meier survival curve was generated, and the log-rank statistics test was rendered. Statistical analyses were performed using commercially available Prism 8 software (GraphPad), and $P < 0.05$ was considered statistically significant.

Supplementary Figures and Figure Legends

Figure S1

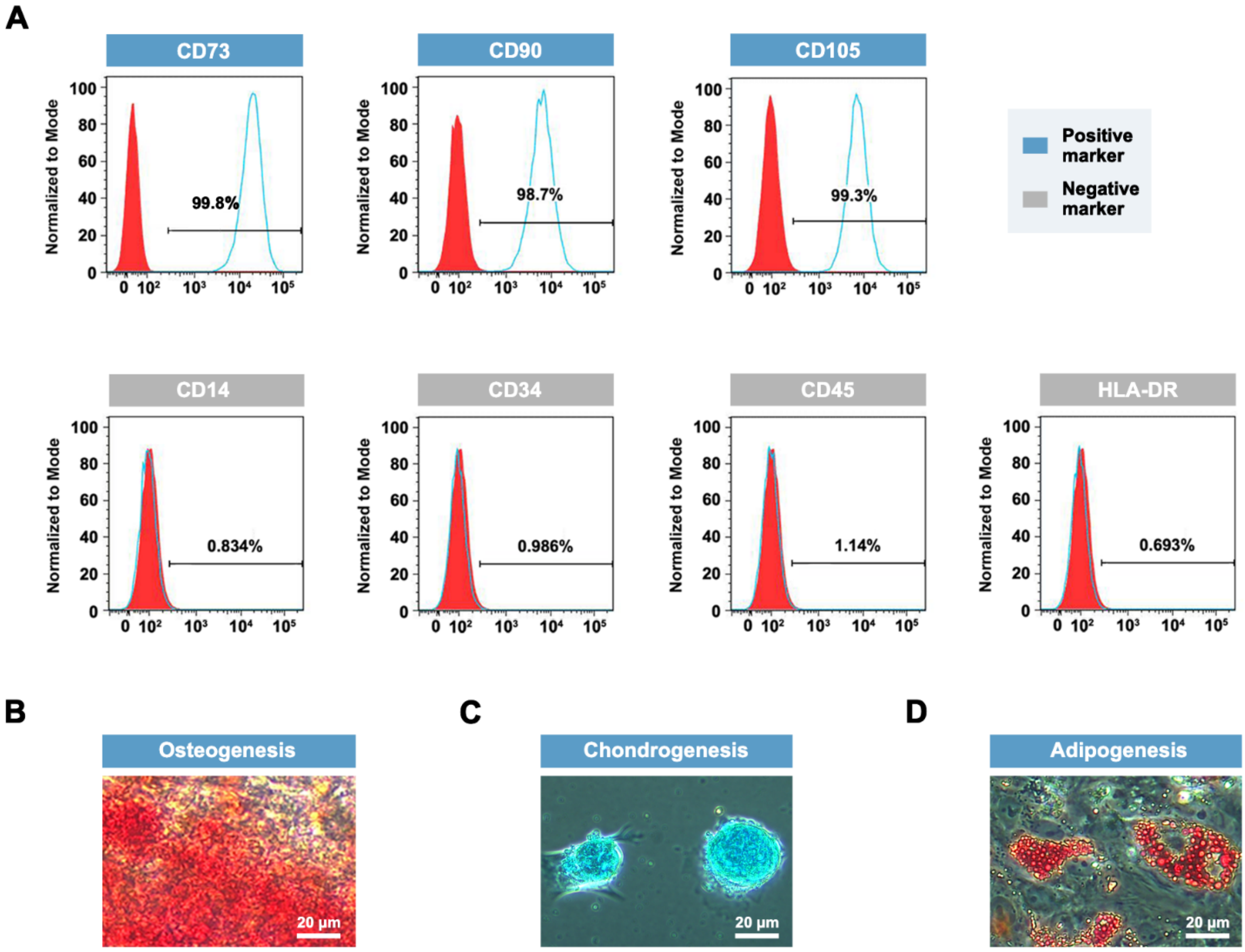

Figure S1. Verification with mesenchymal stem cell (MSC) profiles. (**A**) Mouse bone marrow-derived MSCs were isolated from the tibia and femoral marrow compartments and stained positive for the MSC-specific markers CD73, CD90, and CD105, and negative for CD14, CD34, CD45, and HLA-DR through flow cytometry (FCM) analysis. (**B** to **D**) To verify cell multipotency, the osteogenic, chondrogenic, and adipogenic capacity of MSCs were identified. (**B**) Representative images of osteogenic MSCs stained with Alizarin Red S for calcium, (**C**) chondrogenic MSCs stained with Alcian Blue for acid mucopolysaccharide, and (**D**) adipogenic MSCs stained with Oil Red O for lipid. Scale bar: 20 μm.

Figure S2

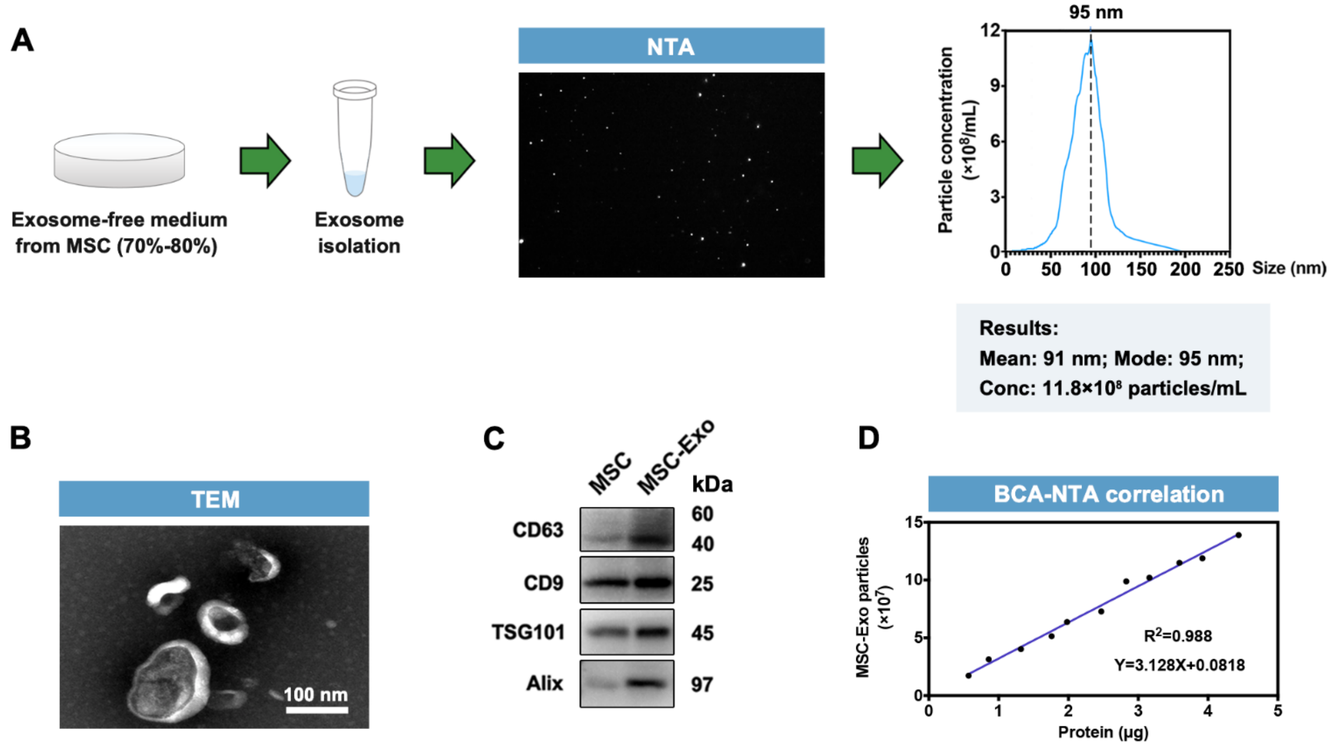

Figure S2. Characterization of exosomes secreted from mouse bone marrow-derived mesenchymal stem cells (MSCs). (**A**) Upon reaching 70%–80% confluency, MSCs were incubated with exosome-free medium and cultured for 48 h. Exosomes were isolated from the culture medium of MSCs and then analyzed by nanoparticle tracking analysis (NTA) to determine their size and concentration. (**B**) Exosome morphology was observed through a transmission electron microscope. Scale bar: 100 nm. (**C**) Representative immunoblots of CD63, CD9, TSG101, and Alix to confirm the presence of exosome markers in isolated exosomes. MSC: MSC lysates, MSC-Exo: MSC-derived exosomes. (**D**) A correlation between protein content (μg) determined by BCA assay and particle number determined by NTA assay was observed.

Figure S3

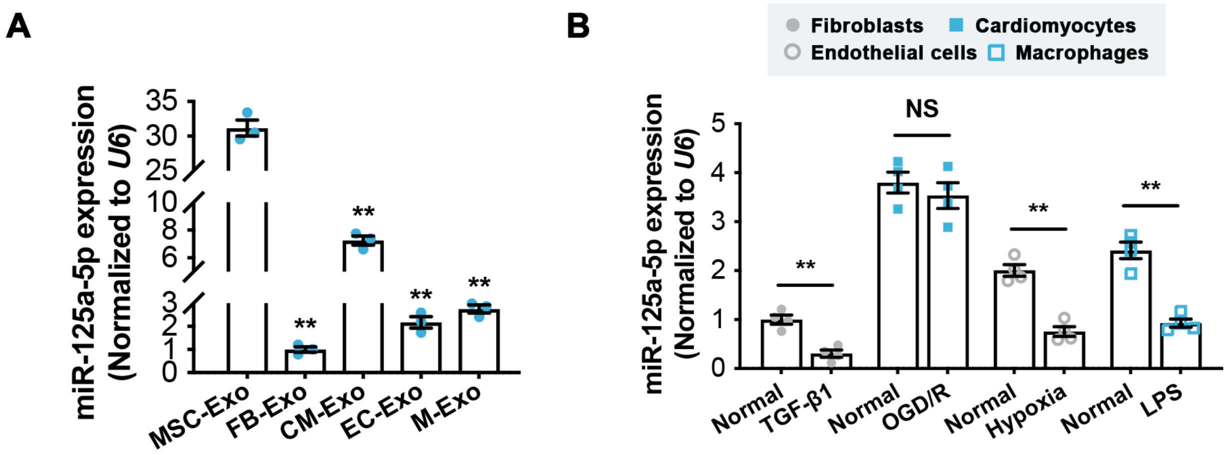

Figure S3. miR-125a-5p expression is enriched in MSC-Exos and decreased in injury stimulated fibroblasts, endothelial cells, and macrophages. (**A**) RT-qPCR quantification of miR-125a-5p in the exosomes obtained from MSCs (MSC-Exo), cardiac fibroblasts (FB-Exo), cardiomyocytes (CM-Exo), endothelial cells (EC-Exo), and macrophages (M-Exo) (n = 3 independent experiments; $^{**}P < 0.01$ versus MSC-Exo group). (**B**) RT-qPCR quantification of miR-125a-5p levels in fibroblasts, cardiomyocytes, endothelial cells, and macrophages treated without or with transforming growth factor-beta1 (TGF-β1), oxygen-glucose deprivation/recovery (OGD/R), hypoxia, or lipopolysaccharide (LPS) stimulation (n = 4 independent experiments). Statistical analysis was performed by one-way ANOVA followed by Tukey's post hoc test in (**A**) and Student's t-test in (**B**). $^{**}P < 0.01$, NS: No significance.

Figure S4

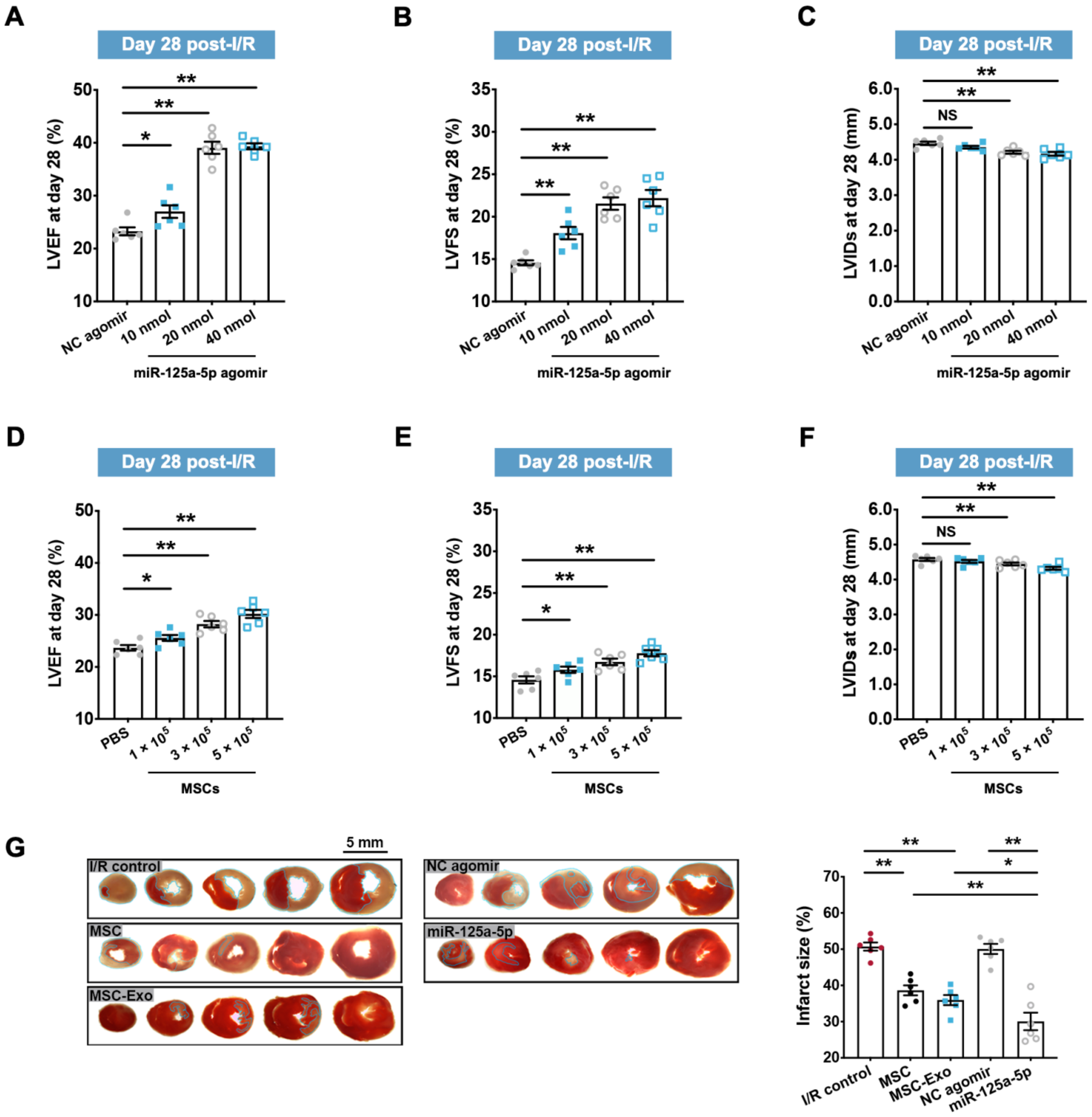
A
Day 28 post-I/R
LVEF at day 28 (%)
NC agomir
10 nmol
20 nmol
40 nmol
miR-125a-5p agomir
B
Day 28 post-I/R
LVFS at day 28 (%)
NC agomir
10 nmol
20 nmol
40 nmol
miR-125a-5p agomir
C
Day 28 post-I/R
LVIDs at day 28 (mm)
NS
NC agomir
10 nmol
20 nmol
40 nmol
miR-125a-5p agomir
D
Day 28 post-I/R
LVEF at day 28 (%)
PBS
1 × 10^5
3 × 10^5
5 × 10^5
MSCs
E
Day 28 post-I/R
LVFS at day 28 (%)
PBS
1 × 10^5
3 × 10^5
5 × 10^5
MSCs
F
Day 28 post-I/R
LVIDs at day 28 (mm)
NS
PBS
1 × 10^5
3 × 10^5
5 × 10^5
MSCs
G
5 mm
I/R control
MSC
MSC-Exo
NC agomir
miR-125a-5p
Infarct size (%)
I/R control
MSC
MSC-Exo
NC agomir
miR-125a-5p

Figure S4. Effects of miR-125a-5p agomir or MSC administration at different doses on myocardial function recovery in myocardial ischemia/reperfusion (I/R) mice. (**A**–**C**) On day 28 post-myocardial I/R, cardiac function, including (**A**) left ventricular (LV) ejection fractions (LVEF), (**B**) LV fractional shortening (LVFS), and (**C**) LV internal diameter at the end-systole (LVIDs), was determined in mice treated with 10, 20, or 40 nmol miR-125a-5p agomir via echocardiography (*n* = 6 mice per group). (**D**–**F**) On day 28 post-myocardial I/R, the (**D**) LVEF, (**E**) LVFS, and (**F**) LVIDs were also measured in mice treated with 1×10^5, 3×10^5, or 5×10^5 MSCs (*n* = 6 mice per group). (**G**) Three days after myocardial I/R, the infarct size (%) of the mouse hearts was calculated by dividing the infarct area (blue dotted) by the total LV area in TTC-stained slices from the apex to base (*n* = 6 mice per group). Statistical analysis was performed by one-way ANOVA followed by Tukey's post hoc test. $^{*}P < 0.05$ and $^{**}P < 0.01$, NS: No significance.

Figure S5

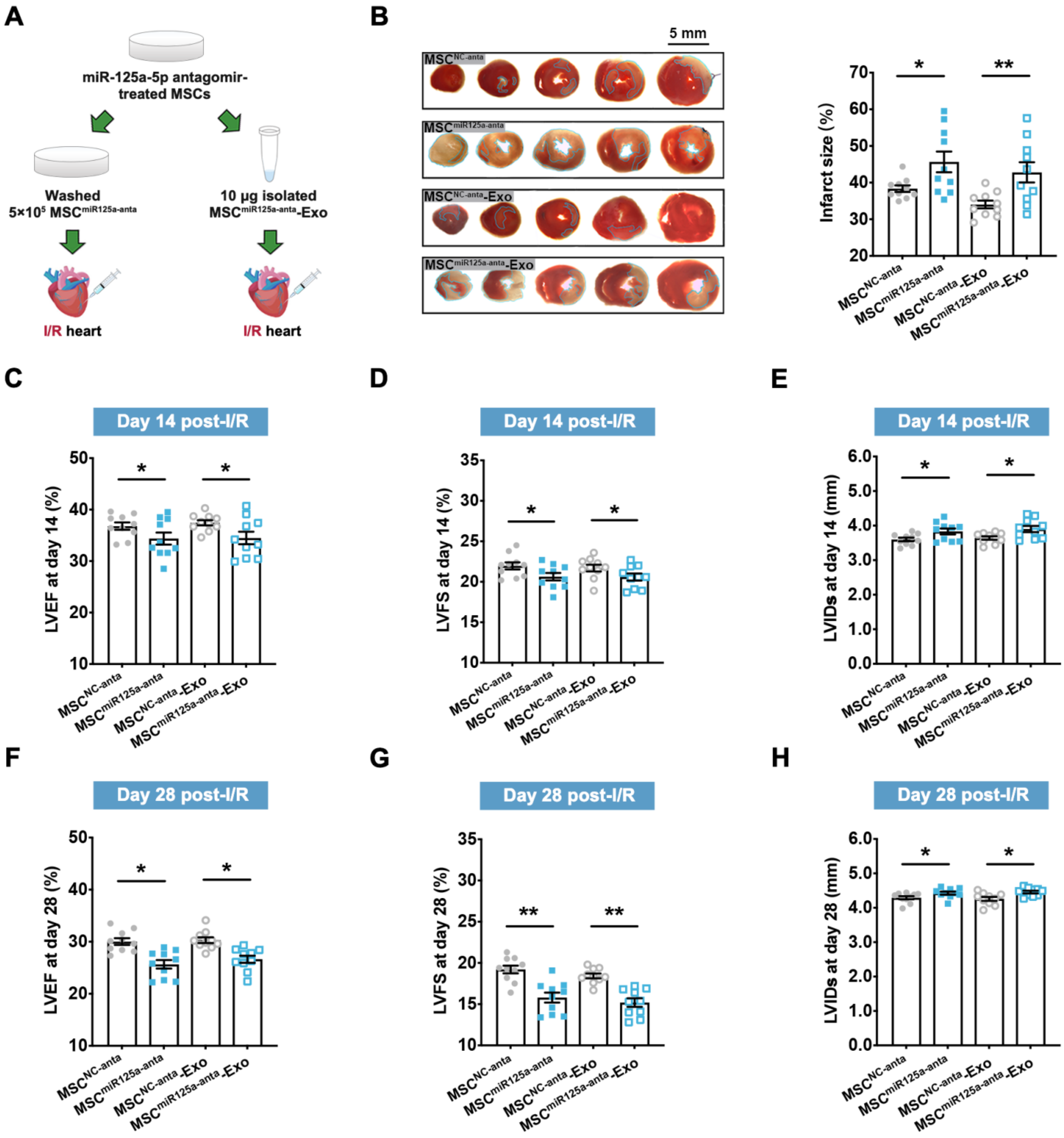

A
miR-125a-5p antagomir-treated MSCs
Washed 5×10⁵ MSC^miR125a-anta
10 µg isolated MSC^miR125a-anta-Exo
I/R heart
I/R heart
B
5 mm
MSC^NC-anta
MSC^miR125a-anta
MSC^NC-anta-Exo
MSC^miR125a-anta-Exo
Infarct size (%)
C
Day 14 post-I/R
LVEF at day 14 (%)
D
Day 14 post-I/R
LVFS at day 14 (%)
E
Day 14 post-I/R
LVIDs at day 14 (mm)
F
Day 28 post-I/R
LVEF at day 28 (%)
G
Day 28 post-I/R
LVFS at day 28 (%)
H
Day 28 post-I/R
LVIDs at day 28 (mm)

Figure S5. miR-125a-5p inhibition partially abolishes the cardioprotection of mesenchymal stem cell (MSC) and MEC-Exo treatments in mouse I/R heart. (**A**) MSCs (5×10^5) were transfected with 200 nM mmu-miR-125a-5p antagomir ($MSC^{miR125a\text{-}anta}$) or 200 nM negative control antagomir ($MSC^{NC\text{-}anta}$), and exosomes were isolated from $MSC^{miR125a\text{-}anta}$ or $MSC^{NC\text{-}anta}$. Injection of these MSCs or MSC-Exos into the mouse heart was performed at the onset of reperfusion. (**B**) Three days after intramyocardial injection of transfected-MSCs or exosomes, the infarct size (%) of I/R hearts was calculated by dividing the infarct area (blue dotted) by the total LV area in five TTC-stained slices from apex to base. (**C–E**) On day 14 post-myocardial I/R, cardiac function, including (**C**) LVEF, (**D**) LVFS, and (**E**) LVIDs, was determined. (**F–H**) On day 28, the (**F**) LVEF, (**G**) LVFS, and (**H**) LVIDs were also measured. $n = 10$ mice per group. Statistical analysis was performed by Student's t test. $^{*}P < 0.05$ and $^{**}P < 0.01$.

Figure S6

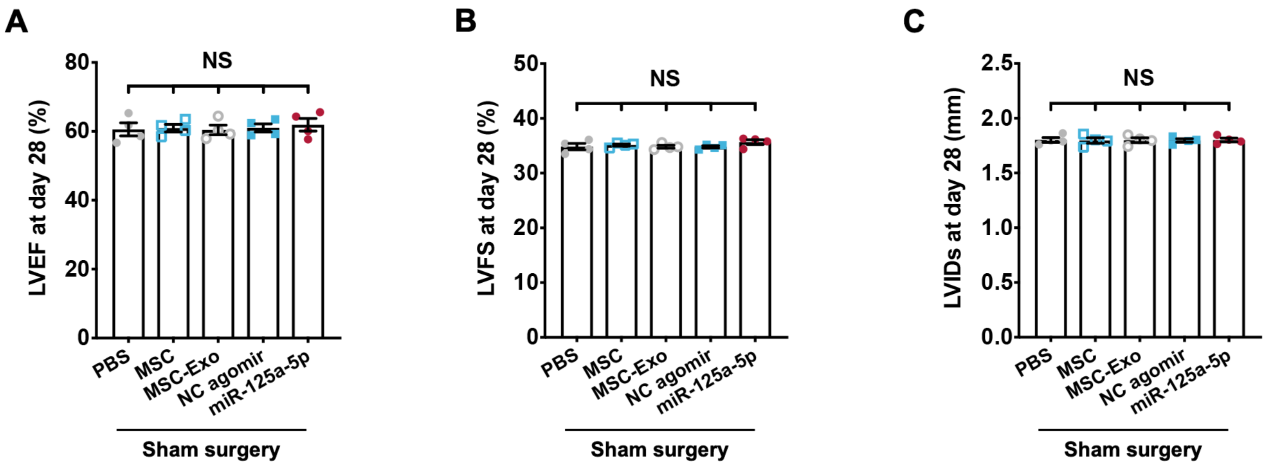

Figure S6. MSC, MSC-Exo, and miR-125a-5p agomir treatments had no significant effect on cardiac function in the sham-operated mice. (**A**–**C**) The (**A**) LVEF, (**B**) LVFS, and (**C**) LVIDs were determined on day 28 post-sham surgery ($n = 4$ mice per group). Statistical analysis was performed by one-way ANOVA followed by Tukey's post hoc test. NS: No significance.

Figure S7

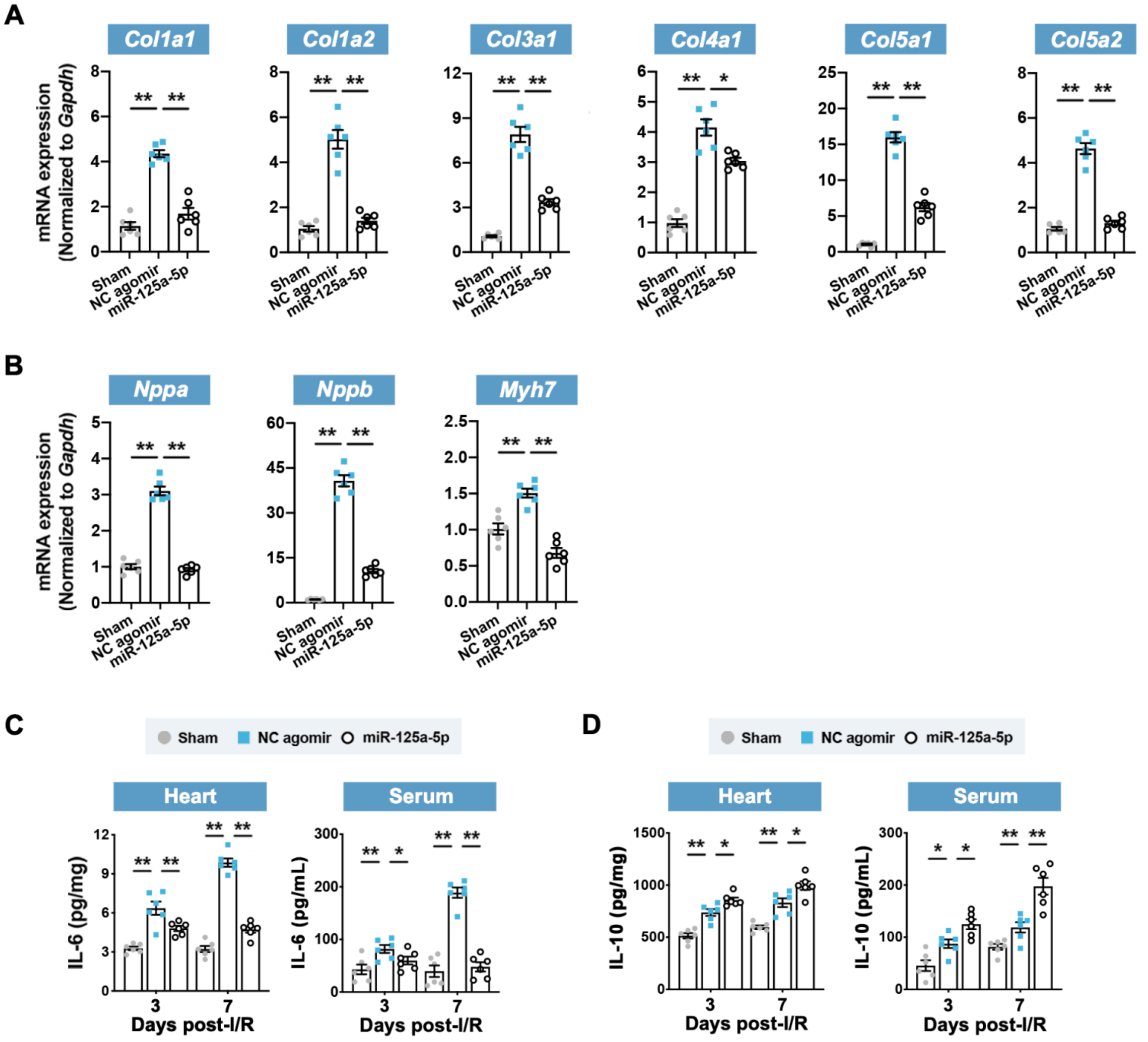

Figure S7. Treatment of miR-125a-5p agomir alleviates the myocardial remodeling-related gene expression levels and inflammatory factor protein content in myocardial I/R mice. (**A** and **B**) Quantification of mRNA expression levels of (**A**) collagen genes (*Col1a1*, *Clo1a2*, *Col3a1*, *Col4a1*, *Col5a1*, and *Col5a2*) and (**B**) myocyte hypertrophy-related genes (*Nppa*, *Nppb*, and *Myh7*) on day 28 post-myocardial I/R. (**C** and **D**) On day 3 and 7 after cardiac I/R, (**C**) IL-6, and (**D**) IL-10 protein concentrations in heart tissue and serum were determined using the corresponding ELISA kits (n = 6 mice per group). Statistical analysis was performed using one-way ANOVA followed by Tukey's post hoc test in (**A** and **B**) and two-way ANOVA followed by Tukey's post hoc test in (**C** and **D**). $^{*}P < 0.05$ and $^{**}P < 0.01$.

Figure S8

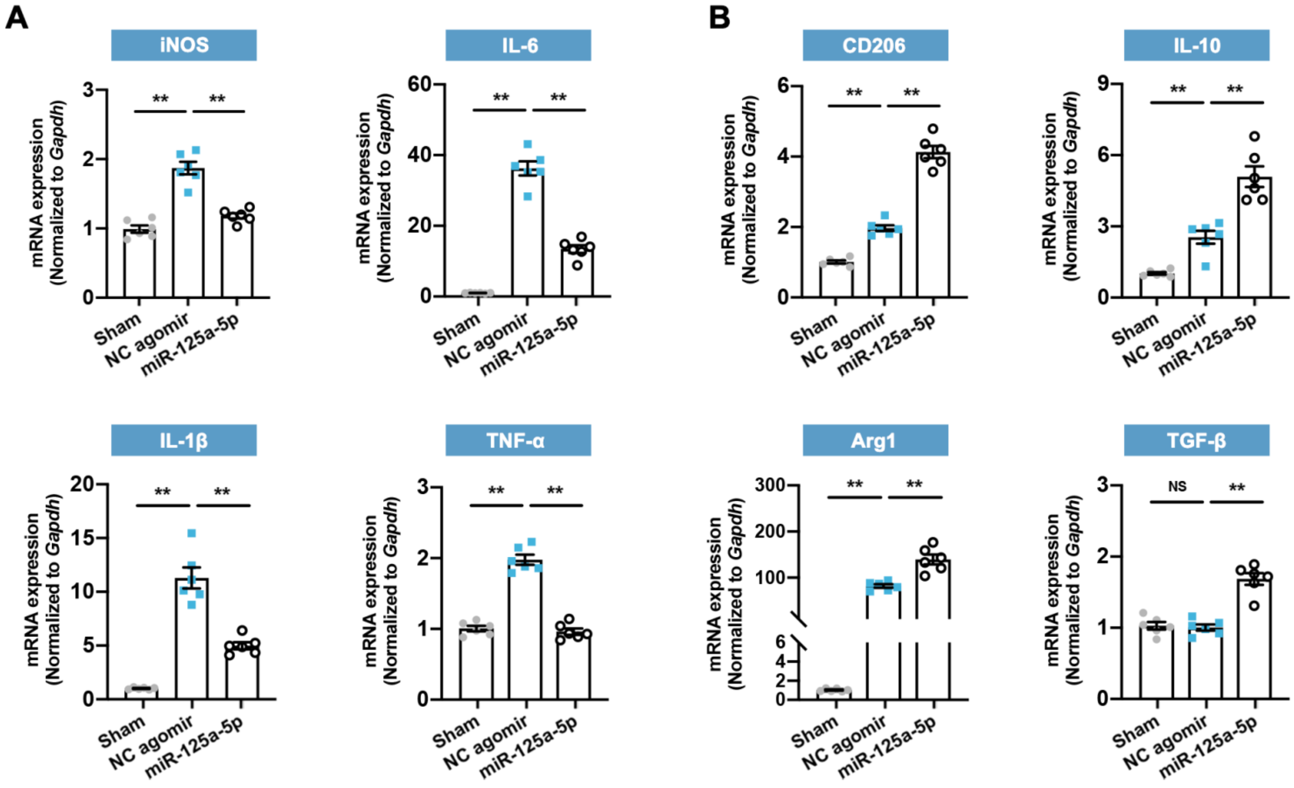

Figure S8. Delivery of miR-125a-5p agomir alters the mRNA expression levels of M1 and M2 macrophage markers and secretory cytokines in mouse I/R hearts. (**A**) Quantification of the gene expression of iNOS and pro-inflammatory cytokines (IL-6, IL-1β, and tumor necrosis factor-alpha [TNF-α]) in the border zone at day 3 post-myocardial I/R. (**B**) Quantification of the gene expression of CD206 and anti-inflammatory cytokines (IL-10, arginase 1 [Arg1], and TGF-β) in the border zone (n = 6 mice per group). Statistical analysis was performed by one-way ANOVA followed by Tukey's post hoc test. $^{**}P < 0.01$, NS: No significance.

Figure S9

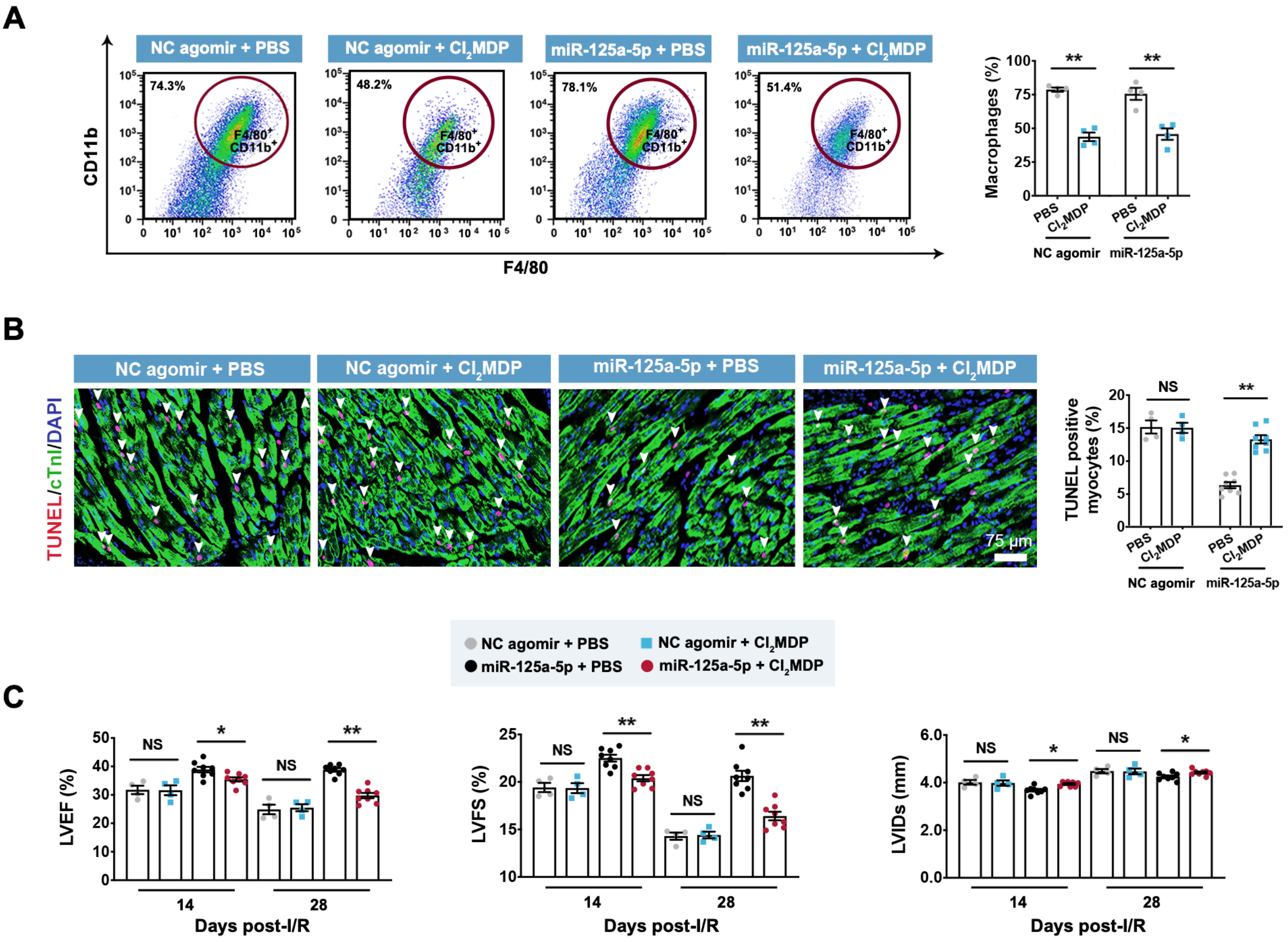

Figure S9. Cardioprotective effects of miR-125a-5p agomir are associated with macrophage activity in cardiac I/R mice. (**A**) Depletion of macrophages in the mouse I/R myocardium was achieved by injection of dichloromethylene diphosphonate liposomes (Cl_2MDP-Lipo), with phosphate buffered saline (PBS) liposomes (PBS-Lipo) used as a control. Flow cytometry (FCM) analysis of macrophages (F4/80$^+$CD11b$^+$) from the hearts of mice on day 3 post-myocardial I/R (n = 4 mice per group). (**B**) Quantitative assessment of TUNEL staining to evaluate the TUNEL-positive cardiomyocytes in the border zone on day 3 post-myocardial I/R. Scale bar: 75 μm. (**C**) Echocardiography for assessing the LVEF, LVFS, and LVIDs on days 14 and 28 post-myocardial I/R (n = 4–8 mice per group). Statistical analysis was performed by Student's t-test in (**A** and **B**) and two-way ANOVA followed by Tukey's post hoc test in (**C**). $^*P < 0.05$ and $^{**}P < 0.01$, NS: No significance.

Figure S10

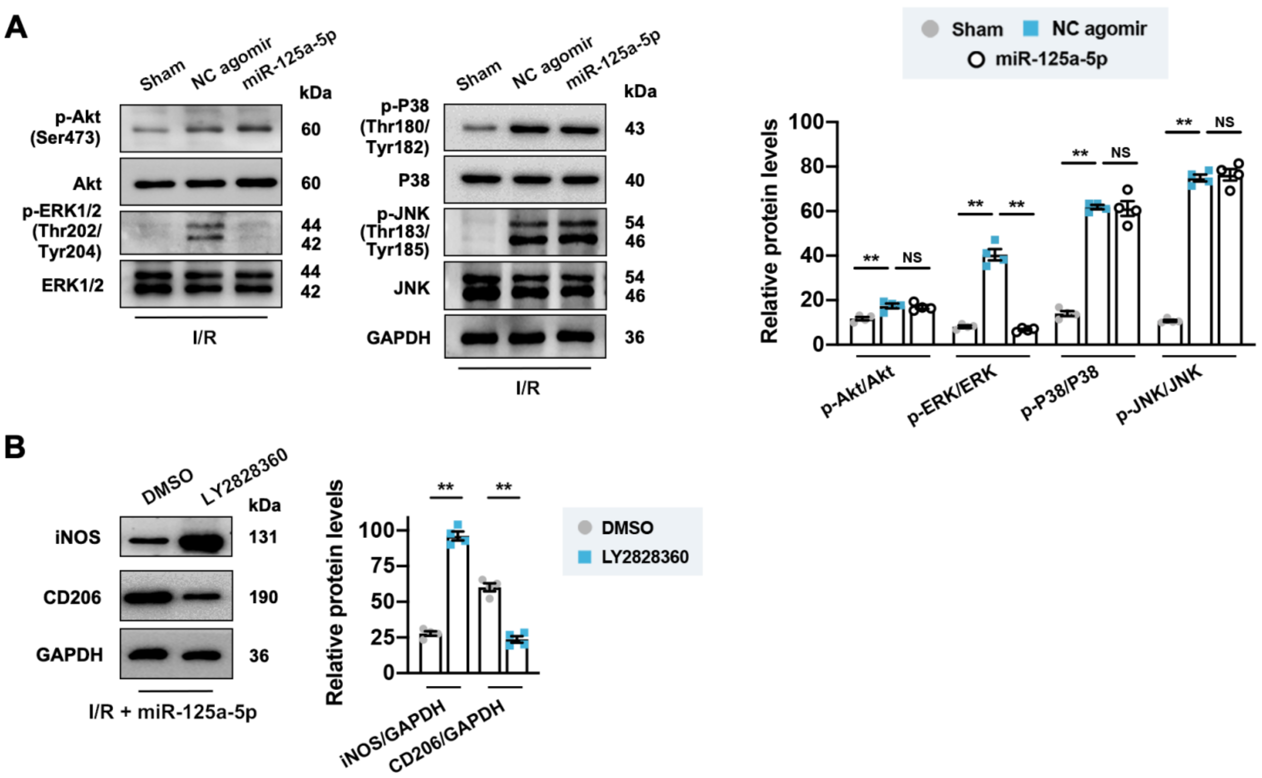

Figure S10. miR-125a-5p regulates macrophage polarization by inhibiting ERK1/2 signaling pathway activation in the I/R myocardium of mice. (**A**) On day 3 post-myocardial I/R, heart tissue was collected for western blotting. Representative immunoblots (left) and quantification (right) for Akt signaling pathway and MAPK-associated signaling cascades, including ERK1/2, P38, and JNK pathways. (**B**) ERK1/2 activator, LY2828360 or DMSO were injected intraperitoneally into the miR-125a-5p mice, and the representative immunoblots (left) and quantification (right) for iNOS and CD206 protein levels on day 3 post-myocardial I/R are shown (n = 4 mice per group). Statistical analysis was performed by one-way ANOVA followed by Tukey's post hoc test in (**A**) and Student's t-test in (**B**). $^{**}P < 0.01$, NS: No significance.

Figure S11

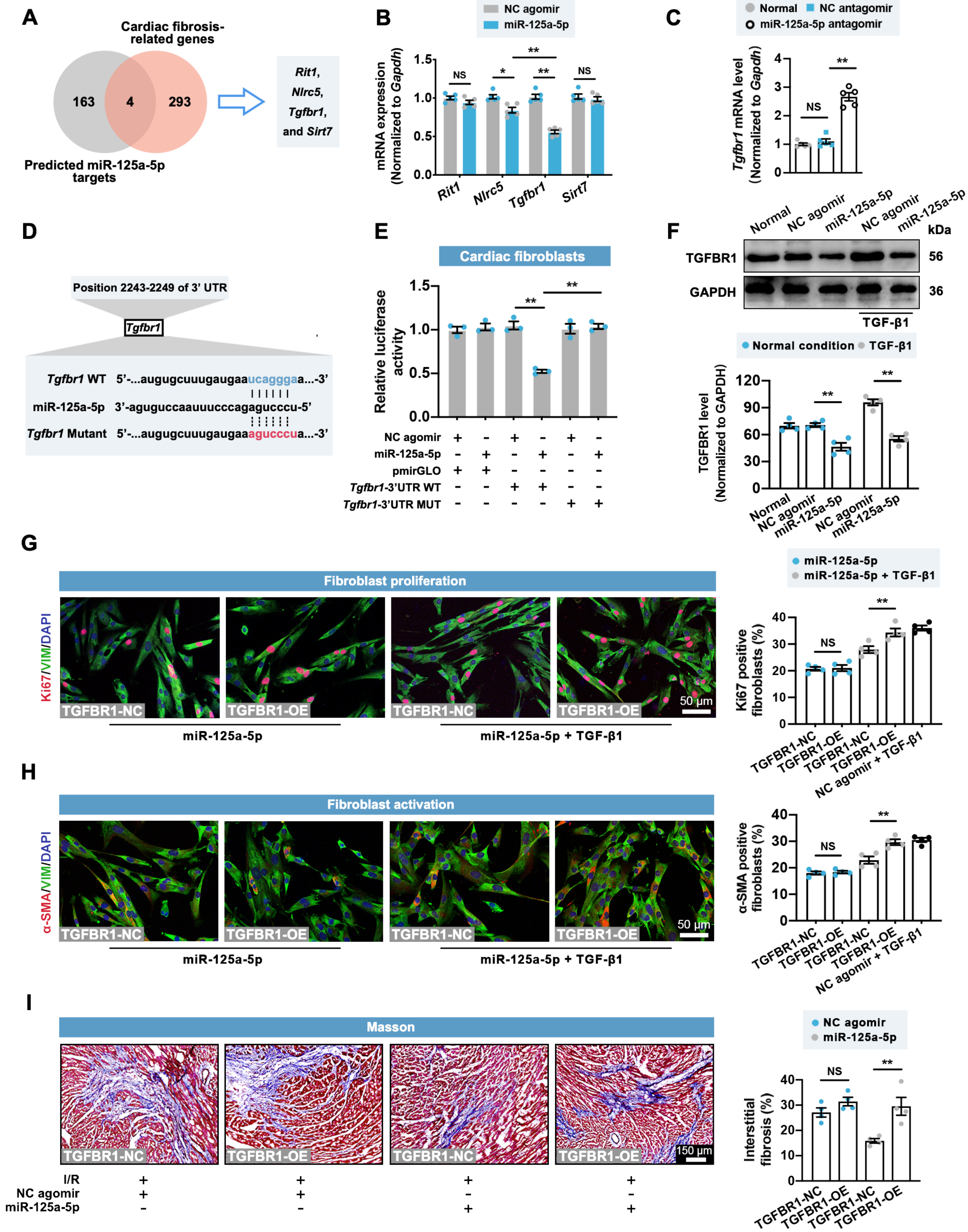

A
Cardiac fibrosis-related genes
163
4
293
Predicted miR-125a-5p targets
Rit1, Nlrc5, Tgfbr1, and Sirt7
B
NC agomir
miR-125a-5p
mRNA expression (Normalized to Gapdh)
Rit1
Nlrc5
Tgfbr1
Sirt7
C
Normal
NC antagomir
miR-125a-5p antagomir
Tgfbr1 mRNA level (Normalized to Gapdh)
D
Position 2243-2249 of 3' UTR
Tgfbr1
Tgfbr1 WT 5'-...augugcuuugaugaaucagggaa...-3'
miR-125a-5p 3'-agugucaauuucccagagucccu-5'
Tgfbr1 Mutant 5'-...augugcuuugaugaaaguccuua...-3'
E
Cardiac fibroblasts
Relative luciferase activity
NC agomir
miR-125a-5p
pmirGLO
Tgfbr1-3'UTR WT
Tgfbr1-3'UTR MUT
F
Normal
NC agomir
miR-125a-5p
kDa
TGFBR1
56
GAPDH
36
TGF-β1
Normal condition
TGF-β1
TGFBR1 level (Normalized to GAPDH)
G
Fibroblast proliferation
Ki67/VIM/DAPI
TGFBR1-NC
TGFBR1-OE
50 μm
miR-125a-5p
miR-125a-5p + TGF-β1
Ki67 positive fibroblasts (%)
NC agomir + TGF-β1
H
Fibroblast activation
α-SMA/VIM/DAPI
α-SMA positive fibroblasts (%)
I
Masson
150 μm
I/R
NC agomir
Interstitial fibrosis (%)

Figure S11. miR-125a-5p agomir regulates fibroblast proliferation and activation under the TGF-β1 condition via acting on *Tgfbr1*. (**A**) The 167 miR-125a-5p target genes predicted from online databases and 297 cardiac fibrosis-related genes predicted from Disgenet were intersected to obtain four candidate genes. (**B**) The mRNA expression levels of *Rit1*, *Nlrc5*, *Tgfbr1*, and *Sirt7* were detected by RT-qPCR in cardiac fibroblasts transfected with miR-125a-5p agomir or NC agomir. (**C**) The mRNA expression level of *Tgfbr1* was detected in normal fibroblasts and in fibroblasts transfected with miR-125a-5p antagomir or NC antagomir (n = 5 independent experiments). (**D**) A WT or mutant dual-luciferase reporter plasmid was constructed according to the predicted binding sequence in 3′ UTR of *Tgfbr1* (blue) or mutant sequence (red), respectively. (**E**) Luciferase activity was determined in cardiac fibroblasts transfected with WT or mutant reporter plasmids and 100 nM miR-125a-5p agomir or NC agomir (n = 3 independent experiments). (**F**) The protein expression of TGF-β receptor-1 (TGFBR1) was assessed by western blotting in normal, normal + NC agomir, normal + miR-125a-5p agomir, TGF-β1 + NC agomir, and TGF-β1 + miR-125a-5p agomir groups. (**G** and **H**) To overexpress TGFBR1 in cardiac fibroblasts, cells were co-cultured with TGFBR1-OE lentiviruses (1×10^8 TU/mL) for 24 h, and TGFBR1-NC lentiviruses were used as a control. Cardiac fibroblasts were then treated with 100 nM miR-125a-5p agomir and some were treated with 20 ng/mL TGF-β1 for 24 h. Representative images and quantitative analysis of (**G**) Ki67 and (**H**) α-SMA positive cardiac fibroblasts. Scale bar: 50 μm (n = 4 independent experiments). (**I**) Quantitative assessment and representative images of Masson's trichrome staining in the border zone of TGFBR1-NC or TGFBR1-OE lentivirus-treated I/R myocardium. Scale bar: 100 μm (n = 4 mice per group). Statistical analysis was performed by one-way ANOVA followed by Tukey's post hoc test in (**B**–**E**) and Student's t-test in (**F**–**I**). $^{*}P < 0.05$ and $^{**}P < 0.01$, NS: No significance.

Figure S12

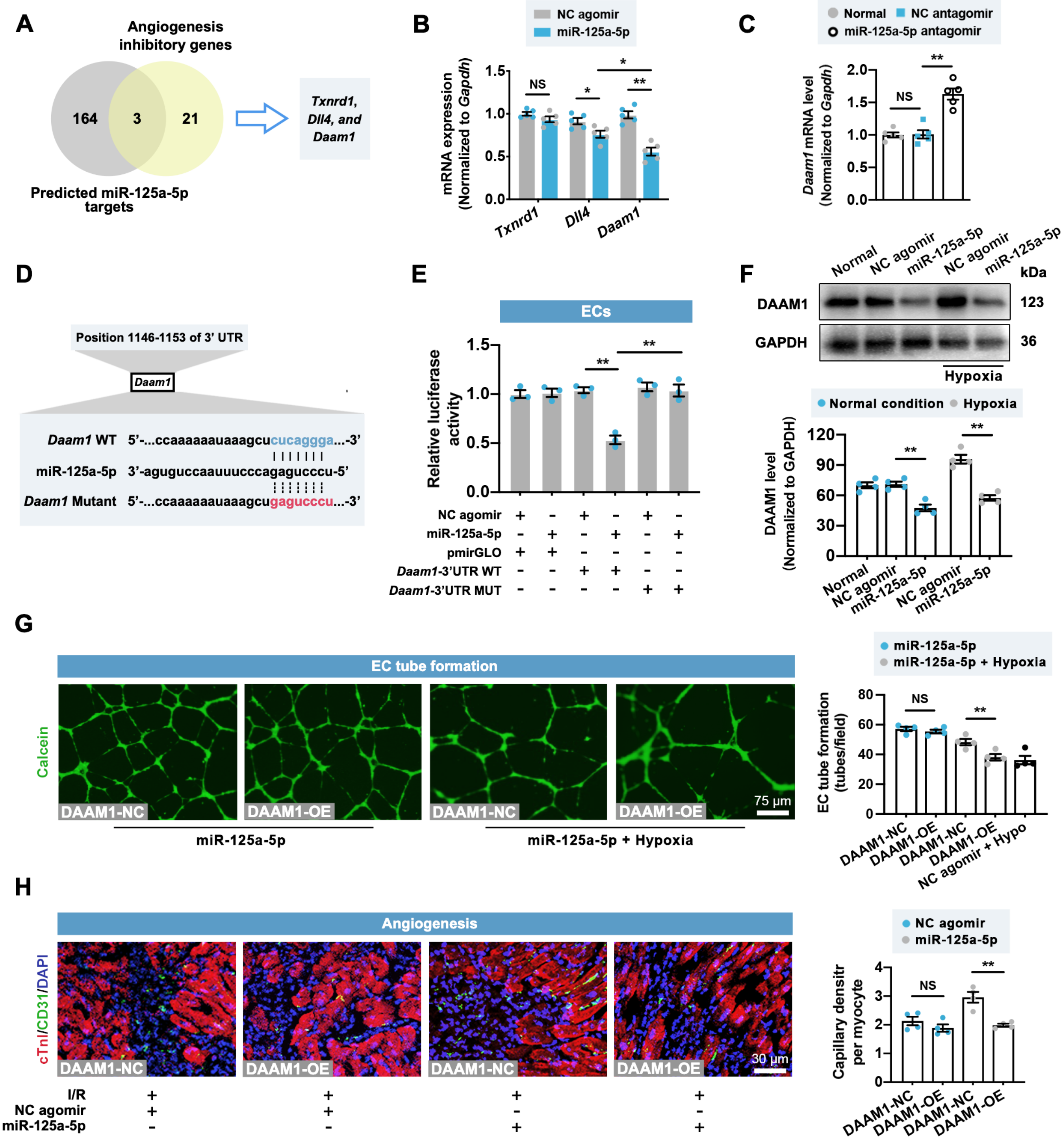

A
Angiogenesis inhibitory genes
164
3
21
Predicted miR-125a-5p targets
Txnrd1, Dll4, and Daam1
B
NC agomir
miR-125a-5p
mRNA expression (Normalized to Gapdh)
Txnrd1
Dll4
Daam1
NS
C
Normal
NC antagomir
miR-125a-5p antagomir
Daam1 mRNA level (Normalized to Gapdh)
D
Position 1146-1153 of 3' UTR
Daam1
Daam1 WT 5'-...ccaaaaaaauaaagcucucaggga...-3'
miR-125a-5p 3'-aguguccaauuucccagagucccu-5'
Daam1 Mutant 5'-...ccaaaaaaauaaagcugagucccu...-3'
E
ECs
Relative luciferase activity
NC agomir
miR-125a-5p
pmirGLO
Daam1-3'UTR WT
Daam1-3'UTR MUT
F
Normal
NC agomir
miR-125a-5p
kDa
DAAM1
GAPDH
123
36
Hypoxia
Normal condition
DAAM1 level (Normalized to GAPDH)
G
EC tube formation
Calcein
DAAM1-NC
DAAM1-OE
75 μm
miR-125a-5p
miR-125a-5p + Hypoxia
EC tube formation (tubes/field)
NC agomir + Hypo
H
Angiogenesis
cTnI/CD31/DAPI
30 μm
I/R
Capillary densitr per myocyte

Figure S12. miR-125a-5p agomir improves injured endothelial cell (EC) function by targeting *Daam1*. (**A**) The 167 miR-125a-5p target genes and 24 angiogenesis inhibitory genes were intersected to obtain three candidate genes. (**B**) The mRNA expression levels of *Txnrd1*, *Dll4*, and *Daam1* were assessed in ECs transfected with miR-125a-5p agomir or NC agomir. (**C**) The level of *Daam1* was investigated in normal ECs and ECs transfected with miR-125a-5p antagomir or NC antagomir ($n = 5$ independent experiments). (**D**) A WT or mutant dual-luciferase reporter plasmid was constructed according to the predicted binding sequence in the 3′ UTR of *Daam1* (blue) or mutant sequence (red), respectively. (**E**) Luciferase activity was detected in ECs transfected with WT or mutant reporter plasmids and 100 nM miR-125a-5p agomir or NC agomir ($n = 3$ independent experiments). (**F**) The protein expression of dishevelled associated activator of morphogenesis 1 (DAAM1) was determined via western blotting in the normal, normal + NC agomir, normal + miR-125a-5p agomir, hypoxia + NC agomir, and hypoxia + miR-125a-5p agomir groups. (**G**) To overexpress DAAM1 in ECs, cells were co-cultured with DAAM1-OE lentiviruses (1×10^8 TU/mL) for 24 h, and DAAM1-NC lentiviruses served as a control. Cells were then treated with 100 nM miR-125a-5p agomir, and some were subjected to hypoxia. Representative images and quantitative assessment of EC tube formation. Scale bar: 75 μm ($n = 4$ independent experiments). (**H**) Representative images and quantitative analysis of CD31 staining for the border zone of myocardial I/R mice on day 28. Scale bars: 30 μm ($n = 4$ mice per group). Statistical analysis was performed by one-way ANOVA followed by Tukey's post hoc test in (**B**–**E**) and Student's *t*-test in (**F**–**H**). $^{**}P < 0.01$, NS: No significance.

Figure S13

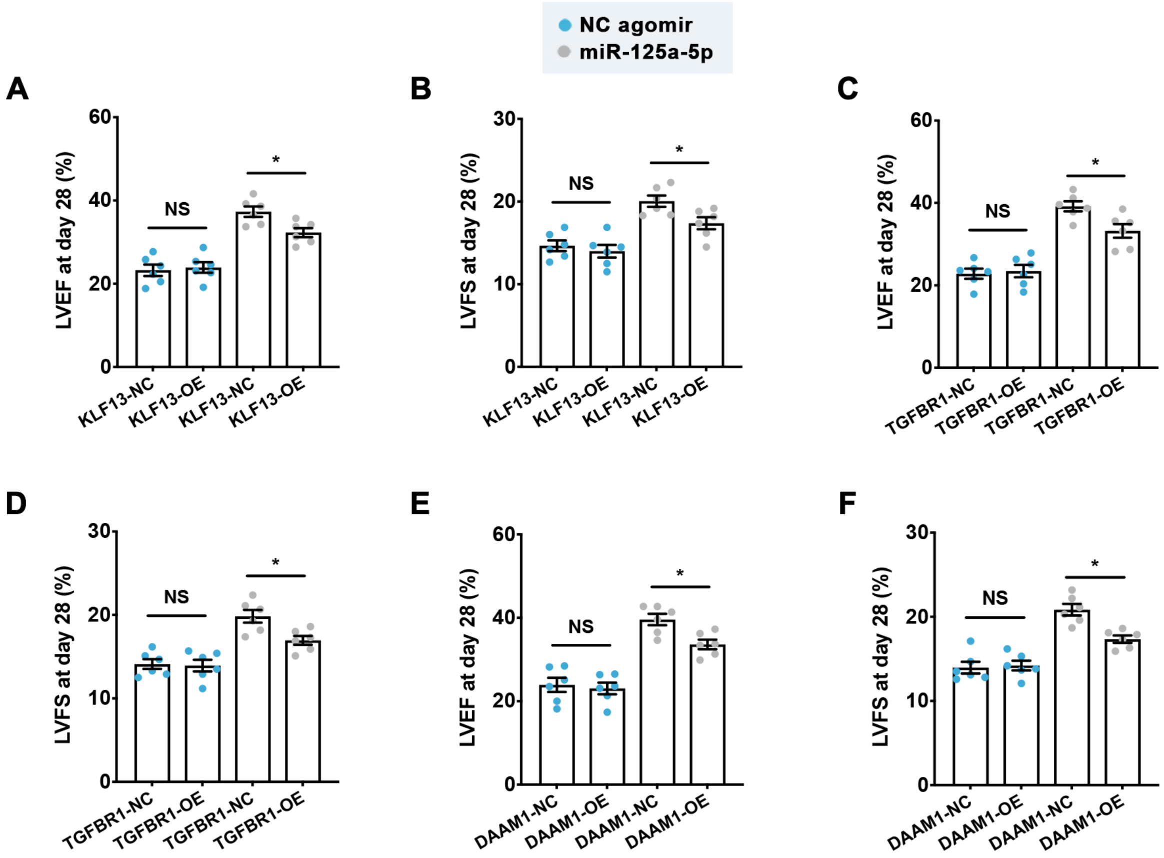

Figure S13. KLF13, TGFBR1, or DAAM1 overexpression partially abrogates the cardioprotective effect of miR-125a-5p in myocardial I/R mice. (**A** and **B**) The (**A**) LVEF and (**B**) LVFS were determined on day 28 post-I/R surgery in KLF13-NC or KLF13-OE lentivirus-treated mice. (**C** and **D**) The (**C**) LVEF and (**D**) LVFS were assessed on day 28 post-I/R surgery in TGFBR1-NC or TGFBR1-OE lentivirus-treated mice. (**E** and **F**) The (**E**) LVEF and (**F**) LVFS were investigated on day 28 post-I/R surgery in DAAM1-NC or DAAM1-OE lentivirus-treated mice (n = 6 mice per group). Statistical analysis was performed using Student's t-test. $^{*}P < 0.05$, NS: No significance.

Figure S14

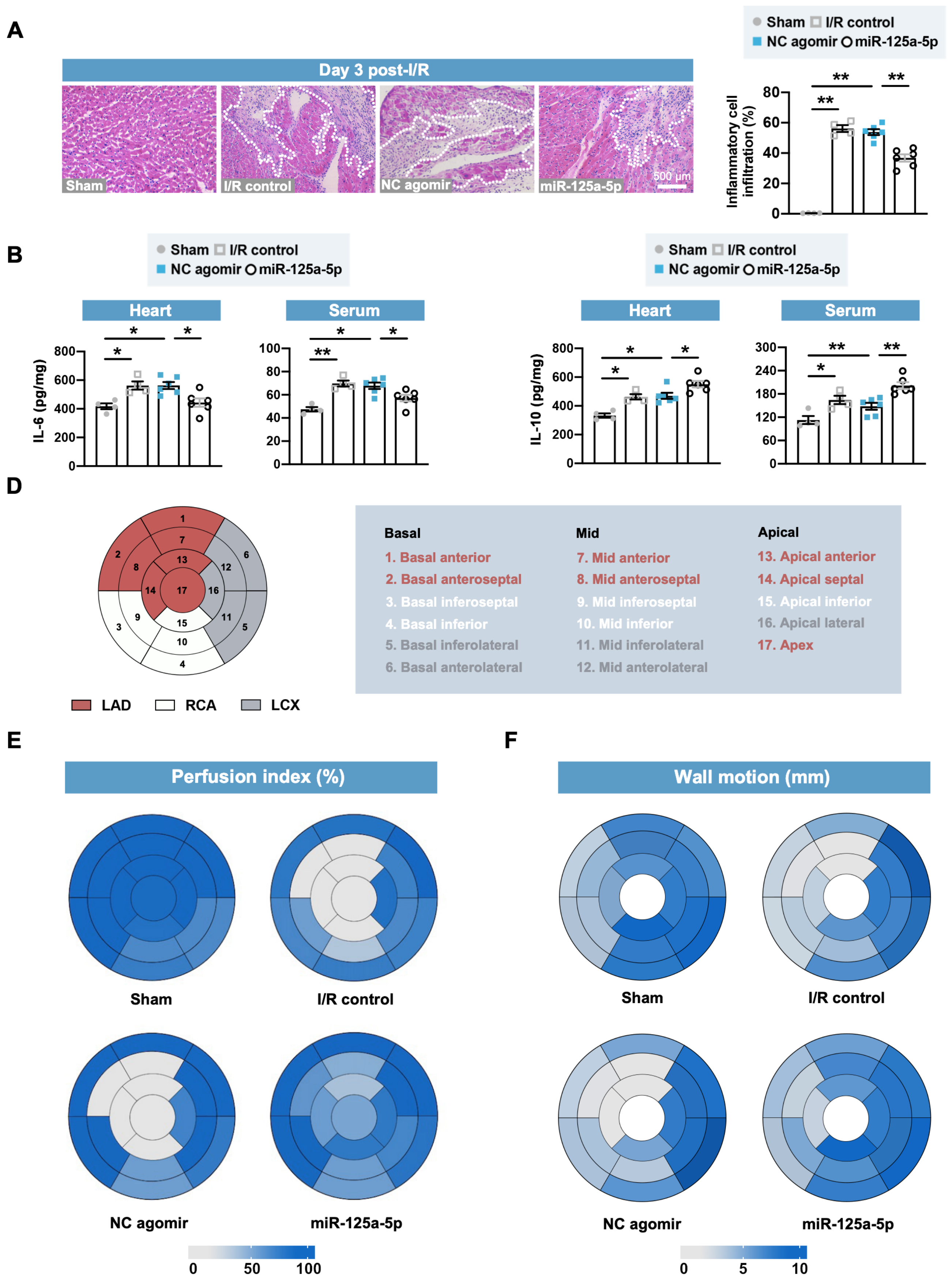

A
Sham
I/R control
NC agomir
miR-125a-5p
Day 3 post-I/R
500 μm
Inflammatory cell infiltration (%)
0
20
40
60
80
**
B
Heart
Serum
IL-6 (pg/mg)
IL-10 (pg/mg)
0
200
400
600
800
20
40
60
80
100
60
120
180
240
300
*
**
D
Basal
1. Basal anterior
2. Basal anteroseptal
3. Basal inferoseptal
4. Basal inferior
5. Basal inferolateral
6. Basal anterolateral
Mid
7. Mid anterior
8. Mid anteroseptal
9. Mid inferoseptal
10. Mid inferior
11. Mid inferolateral
12. Mid anterolateral
Apical
13. Apical anterior
14. Apical septal
15. Apical inferior
16. Apical lateral
17. Apex
LAD
RCA
LCX
E
Perfusion index (%)
Sham
I/R control
NC agomir
miR-125a-5p
0
50
100
F
Wall motion (mm)
Sham
I/R control
NC agomir
miR-125a-5p
0
5
10

Figure S14. hsa-miR-125a-5p delivery decreases cardiac inflammation and improves regional cardiac function in myocardial I/R swine. (**A**) Representative images of H&E staining (left) and quantification of inflammatory cell infiltration (right) in the border zone on day 3 post-myocardial I/R. Scale bar: 500 μm. (**B** and **C**) On day 3 post-myocardial I/R, (**B**) IL-6 and (**C**) IL-10 concentrations in porcine heart tissue and serum were measured (n = 4–6 swine per group). (**D**) Standard classification of myocardial 17-segment regions for MDCT myocardial wall motion and perfusion imaging. The territory of the primary arteries is shown. LAD: Left anterior descending, RCA: Right coronary artery, LCX: Left circumflex artery. (**E** and **F**) Bull's eye diagrams for the absolute values of myocardial segmental (**E**) perfusion (%) and (**F**) wall motion (mm) on day 28 post-cardiac I/R. Statistical analysis was performed by one-way ANOVA followed by Tukey's post hoc test. $^{*}P < 0.05$ and $^{**}P < 0.01$.

Figure S15

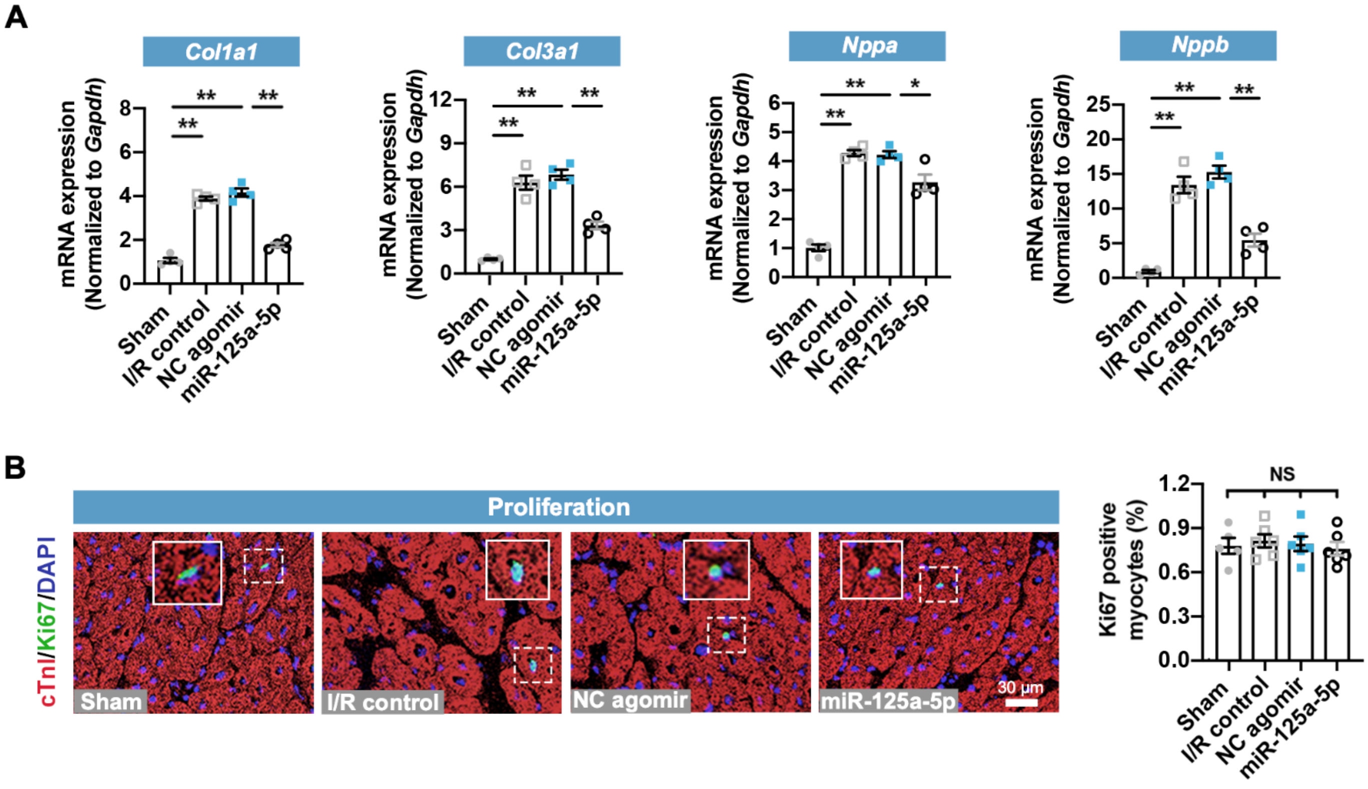

Figure S15. miR-125a-5p therapy reduces the cardiac remodeling related gene expression after myocardial I/R injury in swine. (**A**) Quantification of the mRNA expression levels of collagen genes (*Col1a1* and *Col3a1*) and myocyte hypertrophy-associated genes (*Nppa* and *Nppb*) in hearts of the sham, I/R control, NC agomir, and miR-125a-5p agomir swine on day 28 post-myocardial I/R (n = 4 swine per group). (**B**) Representative images and quantitative assessment of Ki67 staining (proliferation-related marker; frames showing higher-magnification images of the area outlined by white dashed lines) in the border zone on day 28. Scale bars: 30 μm (n = 5–6 swine per group). Statistical analysis was performed by one-way ANOVA followed by Tukey's post hoc test. $^{*}P < 0.05$ and $^{**}P < 0.01$, NS: No significance.

Table S1. Antibodies

Primary antibodies used for FACS analysis

Target/Isotype	Manufacturer/Catalog No.	Dilution
CD73-Alexa Fluor 647/Rat IgG	BD Biosciences/561543	1:20
CD90-FITC/Mouse IgG	BD Biosciences/554894	1:50
CD105-BV421/Rat IgG	BD Biosciences/562760	1:20
CD14-PE/Rat IgG	BD Biosciences/553740	1:20
CD34-PE/Rat IgG	BD Biosciences/551387	1:20
CD45-PE/Rat IgG	BD Biosciences/561087	1:20
HLA-DR-PE/Rat IgG	Miltenyi Biotec/130102896	1:10
F4/80-PE/Rat IgG	BD Biosciences/565410	1:20
CD11b-BV421/Rat IgG	BD Biosciences/562605	1:20
CD206-Alexa Fluor 647/Rat IgG	BD Biosciences/565250	1:20
iNOS-FITC/Mouse IgG	BD Biosciences/610330	1:25
Isotype Control-Alexa Fluor 647/Rat IgG	BD Biosciences/557690	1:20

Primary antibodies used for immunostaining

Target/Isotype	Manufacturer/Catalog No.	Dilution
F4/80/Rat IgG	Abcam/ab6640	1:100
Vimentin/Mouse IgG	Abcam/ab8978	1:200
CD31/Rabbit IgG	Abcam/ab28364	1:100
iNOS-Alexa Fluor 555/Rabbit IgG	Abcam/ab209594	1:50
CD206/Rabbit IgG	Abcam/ab64693	1:500
KLF13/Rabbit IgG	Proteintech/18352-1-AP	1:100
Ki67/Rabbit IgG	Abcam/ab16667	1:300
cTnI/Rabbit IgG	Abcam/ab47003	1:100
cTnI/Goat IgG	Abcam/ab188877	1:100
α-SMA/Rabbit IgG	Abcam/ab124964	1:250

Primary antibodies used for western blotting

Target/Isotype	Manufacturer/Catalog No.	Dilution
CD63/Rabbit IgG	Abcam/ab217345	1:1000
CD9/Rabbit IgG	Abcam/ab223052	1:500
TSG101/Rabbit IgG	Abcam/ab125011	1:1000
Alix/Rabbit IgG	Abcam/ab76608	1:1000
GAPDH/Mouse IgG	Abcam/ab8245	1:1000
KLF13/Rabbit IgG	Abcam/ab190624	1:500
iNOS/Rabbit IgG	Abcam/ab202417	1:1000
CD206/Rabbit IgG	Abcam/ab64693	1:1000
p-Akt (Ser473)/Rabbit IgG	Cell Signaling/4060	1:2000
Akt/Rabbit IgG	Cell Signaling/9272	1:1000
p-ERK1/2 (Thr202/Tyr204)/Rabbit IgG	Cell Signaling/4370	1:1000
ERK1/2/ Rabbit IgG	Cell Signaling/4695	1:1000
p-P38 (Thr180/Tyr182)/Rabbit IgG	Cell Signaling/4511	1:1000
P38/Rabbit IgG	Cell Signaling/8690	1:1000
p-JNK (Thr183/Tyr185)/mouse IgG	Cell Signaling/9255	1:2000
JNK/Rabbit IgG	Cell Signaling/9252	1:1000
TGFBR1/Rabbit IgG	Abcam/ab31013	1:1000
DAAM1/Rabbit IgG	Sigma-Aldrich/SAB2100527	1:500

Secondary antibodies

Target	Manufacturer/Catalog No.	Dilution
Donkey anti-Mouse IgG-Alexa Fluor 488	Jackson Lab/715-545-150	1:100
Donkey anti-Rat IgG-Alexa Fluor 488	Thermo Fisher Scientific /A21208	1:2000
Donkey anti-Rabbit IgG-Alexa Fluor 488	Jackson Lab/711-545-152	1:100
Donkey anti-Rabbit IgG-Cy3	Jackson Lab/711-165-152	1:100
Donkey anti-Goat IgG-Rhodamine	Jackson Lab/705-025-147	1:100
Donkey anti-Goat IgG-Alexa Fluor 647	Jackson Lab/705-605-147	1:100
Goat anti-mouse IgG-HRP	Sigma-Aldrich/A4416	1:4000
Goat anti-rabbit IgG-HRP	Sigma-Aldrich/A0545	1:4000

Table S2. Differentially expressed microRNAs in NGS data

microRNAs	Fold change (log_2) MSC-Exo vs FB-Exo	*P*-value	Up/Down
mmu-miR-125a-5p	4.98	0.000017	Up
mmu-miR-125b-5p	3.91	0.000376	Up
mmu-miR-21	3.06	0.000357	Up
mmu-miR-182	2.97	0.000635	Up
mmu-miR-29	2.89	0.005173	Up
mmu-miR-25-3p	2.27	0.000741	Up
mmu-miR-30	1.95	0.002184	Up
mmu-miR-22	1.54	0.000893	Up
mmu-miR-124	1.26	0.002457	Up
mmu-miR-103-3p	0.99	0.007493	Up
mmu-miR-3473	0.81	0.025134	Up
mmu-miR-378a	0.63	0.039385	Up
mmu-miR-377-3p	0.56	0.026749	Up
mmu-miR-181	0.52	0.038541	Up
mmu-miR-381	−4.85	0.000138	Down
mmu-miR-223	−3.35	0.014958	Down
mmu-miR-379	−2.89	0.008576	Down
mmu-miR-31	−1.73	0.037582	Down
mmu-miR-542-3p	−1.58	0.025492	Down

Table S3. Primers used for RT-qPCR

Gene	Sequence
Mouse *Col1a1* (forward)	5′-GCTCCTCTTAGGGGCCACT-3′
Mouse *Col1a1* (reverse)	5′-ATTGGGGACCCTTAGGCCAT-3′
Mouse *Col1a2* (forward)	5′-GGTGAGCCTGGTCAAACGG-3′
Mouse *Col1a2* (reverse)	5′-ACTGTGTCCTTTCACGCCTTT-3′
Mouse *Col3a1* (forward)	5′-CTGTAACATGGAAACTGGGGAAA-3′
Mouse *Col3a1* (reverse)	5′-CCATAGCTGAACTGAAAACCACC-3′
Mouse *Col4a1* (forward)	5′-CCTGGCACAAAAGGGACGA-3′
Mouse *Col4a1* (reverse)	5′-ACGTGGCCGAGAATTTCACC-3′
Mouse *Col5a1* (forward)	5′-CTTCGCCGCTACTCCTGTTC-3′
Mouse *Col5a1* (reverse)	5′-CCCTGAGGGCAAATTGTGAAAA-3′
Mouse *Col5a2* (forward)	5′-ACAGGTGAAGTGGGATTCTCA-3′
Mouse *Col5a2* (reverse)	5′-CCATAGCACCCATTGGACCA-3′
Mouse *Nppa* (forward)	5′-GTGCGGTGTCCAACACAGAT-3′
Mouse *Nppa* (reverse)	5′-TCCAATCCTGTCAATCCTACCC-3′
Mouse *Nppb* (forward)	5′-GAGGTCACTCCTATCCTCTGG-3′
Mouse *Nppb* (reverse)	5′-GCCATTTCCTCCGACTTTTCTC-3′
Mouse *Myh7* (forward)	5′-ACTGTCAACACTAAGAGGGTCA-3′
Mouse *Myh7* (reverse)	5′-TTGGATGATTTGATCTTCCAGGG-3′
Mouse *Nos2* (forward)	5′-GTTCTCAGCCCAACAATACAAGA-3′
Mouse *Nos2* (reverse)	5′-GTGGACGGGTCGATGTCAC-3′
Mouse *Il6* (forward)	5′-TAGTCCTTCCTACCCCAATTTCC-3′
Mouse *Il6* (reverse)	5′-TTGGTCCTTAGCCACTCCTTC-3′

Mouse *Il1b* (forward)	5′-GCAACTGTTCCTGAACTCAACT-3′
Mouse *Il1b* (reverse)	5′-ATCTTTTGGGGTCCGTCAACT-3′
Mouse *Tnf* (forward)	5′-CCCTCACACTCAGATCATCTTCT-3′
Mouse *Tnf* (reverse)	5′-GCTACGACGTGGGCTACAG-3′
Mouse *Mrc1* (forward)	5′-CTCTGTTCAGCTATTGGACGC-3′
Mouse *Mrc1* (reverse)	5′-CGGAATTTCTGGGATTCAGCTTC-3′
Mouse *Il10* (forward)	5′-GCTCTTACTGACTGGCATGAG-3′
Mouse *Il10* (reverse)	5′-CGCAGCTCTAGGAGCATGTG-3′
Mouse *Arg1* (forward)	5′-CTCCAAGCCAAAGTCCTTAGAG-3′
Mouse *Arg1* (reverse)	5′-AGGAGCTGTCATTAGGGACATC-3′
Mouse *Tgfb1*(forward)	5′-CTCCCGTGGCTTCTAGTGC-3′
Mouse *Tgfb1*(reverse)	5′-GCCTTAGTTTGGACAGGATCTG-3′
Mouse *Klf13* (forward)	5′-CCTGGCCTCAGACAAAGGG-3′
Mouse *Klf13* (reverse)	5′-ATTTCCCGTAAACTTTCTCGCA-3′
Mouse *Ptpn1* (forward)	5′-GGAACTGGGCGGCTATTTACC-3′
Mouse *Ptpn1* (reverse)	5′-CAAAAGGGCTGACATCTCGGT-3′
Mouse *Lif* (forward)	5′-ATTGTGCCCTTACTGCTGCTG-3′
Mouse *Lif* (reverse)	5′-GCCAGTTGATTCTTGATCTGGT-3′
Mouse *Cln6* (forward)	5′-CTCAGTCAACCATCGCCTG-3′
Mouse *Cln6* (reverse)	5′-GCTTGAGGTTCTTGATAATGGGG-3′
Mouse *Tnfaip3* (forward)	5′-GAACAGCGATCAGGCCAGG-3′
Mouse *Tnfaip3* (reverse)	5′-GGACAGTTGGGTGTCTCACATT-3′
Mouse *Rit1* (forward)	5′-GCCACCGATTCCCAGAAGAC-3′
Mouse *Rit1* (reverse)	5′-GATCCCGCATGGCTGTAAACT-3′

Mouse *Nlrc5* (forward)	5′-GTGCCAAACGTCCTTTTCAGA-3′
Mouse *Nlrc5* (reverse)	5′-AGTGAGGAGTAAGCCATGCTC-3′
Mouse *Tgfbr1* (forward)	5′-CAGCTCCTCATCGTGTTGGTG-3′
Mouse *Tgfbr1* (reverse)	5′-GCACATACAAATGGCCTGTCTC-3′
Mouse *Sirt7* (forward)	5′-CAGGTGTCACGCATCCTGAG-3′
Mouse *Sirt7* (reverse)	5′-GCCCGTGTAGACAACCAAGT-3′
Mouse *Txnrd1* (forward)	5′-CCCACTTGCCCCAACTGTT-3′
Mouse *Txnrd1* (reverse)	5′-GGGAGTGTCTTGGAGGGAC-3′
Mouse *Dll4* (forward)	5′-TTCCAGGCAACCTTCTCCGA-3′
Mouse *Dll4* (reverse)	5′-ACTGCCGCTATTCTTGTCCC-3′
Mouse *Daam1* (forward)	5′-AACTTTGCACTTCAGACAATGGA-3′
Mouse *Daam1* (reverse)	5′-CTGGTCCTTTTTCTTGCTACAGT-3′
Mouse *Gapdh* (forward)	5′-AGGTCGGTGTGAACGGATTTG-3′
Mouse *Gapdh* (reverse)	5′-TGTAGACCATGTAGTTGAGGTCA-3′
Porcine *Col1a1* (forward)	5′-CCCGAGGCTCTGAAGGTC-3′
Porcine *Col1a1* (reverse)	5′-CATCAGCACCAGGGTTTCC-3′
Porcine *Col3a1* (forward)	5′-CCTCATTAGTCCCGATGGTT-3′
Porcine *Col3a1* (reverse)	5′-TTTGCAGCCTTGGTTAGGA-3′
Porcine *Nppa* (forward)	5′-TCGTTCTGGTGTTTCAGTTCC-3′
Porcine *Nppa* (reverse)	5′-AGGCATCTTGTCCTCCAAGT-3′
Porcine *Nppb* (forward)	5′-GGTCCAGCAGCTCCTGTATC-3′
Porcine *Nppb* (reverse)	5′-GCTCCTGCTCCTGTTCTTGCA-3′
Porcine *Gapdh* (forward)	5′-ATTGCCCTCAACGACCACT-3′
Porcine *Gapdh* (reverse)	5′-ATGAGGTCCACCACCCTGT-3′

Table S4. Primary efficacy outcomes of cardiac function in mice

Variable	Group	n	14 days	28 days
LVEF (%)	Sham	7	60.5 ± 1.42	60.1 ± 1.09
	I/R control	7	31.4 ± 0.48	23.6 ± 0.71
	MSC	10	36.4 ± 0.67	29.9 ± 0.71
	MSC-Exo	10	36.5 ± 0.54	30.2 ± 0.50
	NC agomir	8	31.4 ± 1.00	23.3 ± 0.71
	miR-125a-5p	11	38.6 ± 0.61	39.1 ± 0.48
LVFS (%)	Sham	7	34.9 ± 0.20	35.2 ± 0.26
	I/R control	7	20.0 ± 0.30	14.4 ± 0.42
	MSC	10	21.9 ± 0.21	18.1 ± 0.21
	MSC-Exo	10	22.4 ± 0.21	17.7 ± 0.22
	NC agomir	8	20.2 ± 0.26	14.6 ± 0.41
	miR-125a-5p	11	22.7 ± 0.26	21.0 ± 0.23
LVIDs (mm)	Sham	7	1.80 ± 0.02	1.80 ± 0.02
	I/R control	7	4.05 ± 0.04	4.57 ± 0.03
	MSC	10	3.65 ± 0.03	4.31 ± 0.04
	MSC-Exo	10	3.69 ± 0.02	4.22 ± 0.03
	NC agomir	8	4.03 ± 0.04	4.53 ± 0.04
	miR-125a-5p	11	3.69 ± 0.03	4.26 ± 0.02
LVIDd (mm)	Sham	7	2.76 ± 0.02	2.77 ± 0.03
	I/R control	7	5.07 ± 0.05	5.34 ± 0.04
	MSC	10	4.67 ± 0.03	5.26 ± 0.05
	MSC-Exo	10	4.75 ± 0.03	5.13 ± 0.03

	NC agomir	8	5.05 ± 0.03	5.31 ± 0.04
	miR-125a-5p	11	4.78 ± 0.04	5.39 ± 0.05
Heart rate (beats/min)	Sham	7	473 ± 13.3	464 ± 25.8
	I/R control	7	458 ± 28.3	452 ± 33.7
	MSC	10	453 ± 31.3	455 ± 29.6
	MSC-Exo	10	459 ± 30.6	451 ± 35.7
	NC agomir	8	449 ± 23.4	452 ± 34.2
	miR-125a-5p	11	465 ± 32.6	461 ± 24.8

Table S5. Primary efficacy outcomes of cardiac function in swine

Variable	Group	n	28 days
LVEF (%)	Sham	5	66.5 ± 2.90
	I/R control	6	45.0 ± 1.61
	NC agomir	8	45.2 ± 1.63
	miR-125a-5p	8	60.6 ± 1.86
Stroke volume (mL)	Sham	5	40.4 ± 3.24
	I/R control	6	32.9 ± 1.72
	NC agomir	8	33.7 ± 1.47
	miR-125a-5p	8	41.2 ± 1.45
LVESV (mL)	Sham	5	20.1 ± 1.40
	I/R control	6	40.1 ± 1.44
	NC agomir	8	41.0 ± 1.87
	miR-125a-5p	8	26.8 ± 1.55
LVEDV (mL)	Sham	5	60.5 ± 2.53
	I/R control	6	73.0 ± 2.14
	NC agomir	8	74.7 ± 2.28
	miR-125a-5p	8	68.0 ± 1.74
CO (L/min)	Sham	5	3.29 ± 0.04
	I/R control	6	2.77 ± 0.02
	NC agomir	8	2.72 ± 0.04
	miR-125a-5p	8	3.24 ± 0.04
Heart rate (beats/min)	Sham	5	81.4 ± 1.58
	I/R control	6	84.2 ± 1.53

	NC agomir	8	80.8 ± 2.87
	miR-125a-5p	8	78.6 ± 2.75

CO: Cardiac output.

Supplementary References

1. Gnecchi M, Melo LG. Bone marrow-derived mesenchymal stem cells: isolation, expansion, characterization, viral transduction, and production of conditioned medium. Methods Mol Biol. 2009; 482: 281-94.
2. Xiao C, Wang K, Xu Y, Hu H, Zhang N, Wang Y, et al. Transplanted Mesenchymal Stem Cells Reduce Autophagic Flux in Infarcted Hearts via the Exosomal Transfer of miR-125b. Circ Res. 2018; 123: 564-78.
3. Gao L, Gregorich ZR, Zhu W, Mattapally S, Oduk Y, Lou X, et al. Large Cardiac Muscle Patches Engineered From Human Induced-Pluripotent Stem Cell-Derived Cardiac Cells Improve Recovery From Myocardial Infarction in Swine. Circulation. 2018; 137: 1712-30.
4. Gao L, Wang L, Wei Y, Krishnamurthy P, Walcott GP, Menasche P, et al. Exosomes secreted by hiPSC-derived cardiac cells improve recovery from myocardial infarction in swine. Sci Transl Med. 2020; 12: eaay1318.
5. Valiente-Alandi I, Potter SJ, Salvador AM, Schafer AE, Schips T, Carrillo-Salinas F, et al. Inhibiting Fibronectin Attenuates Fibrosis and Improves Cardiac Function in a Model of Heart Failure. Circulation. 2018; 138: 1236-52.
6. Lin X, Dhopeshwarkar AS, Huibregtse M, Mackie K, Hohmann AG. Slowly Signaling G Protein-Biased CB2 Cannabinoid Receptor Agonist LY2828360 Suppresses Neuropathic Pain with Sustained Efficacy and Attenuates Morphine Tolerance and Dependence. Mol Pharmacol. 2018; 93: 49-62.
7. Ackers-Johnson M, Li PY, Holmes AP, O'Brien SM, Pavlovic D, Foo RS. A Simplified, Langendorff-Free Method for Concomitant Isolation of Viable Cardiac Myocytes and Nonmyocytes From the Adult Mouse Heart. Circ Res. 2016; 119: 909-20.
8. Pino PA, Cardona AE. Isolation of brain and spinal cord mononuclear cells using percoll gradients. J Vis Exp. 2011; e2348.
9. Gao R, Wang L, Bei Y, Wu X, Wang J, Zhou Q, et al. Long Noncoding RNA Cardiac Physiological Hypertrophy-Associated Regulator Induces Cardiac Physiological Hypertrophy and Promotes Functional Recovery After Myocardial Ischemia-Reperfusion Injury. Circulation. 2021; 144: 303-17.
10. Hafeez Y, Quintanilla Rodriguez BS, Ahmed I, Grossman SA. Paroxysmal Supraventricular Tachycardia. StatPearls. Treasure Island (FL); 2021.
11. Kaplan J, Lala V. Paroxysmal Atrial Tachycardia. StatPearls. Treasure Island (FL); 2021.
12. Pedersen CT, Kay GN, Kalman J, Borggrefe M, Della-Bella P, Dickfeld T, et al. EHRA/HRS/APHRS expert consensus on ventricular arrhythmias. Europace. 2014; 16: 1257-83.
13. Yokota T, McCourt J, Ma F, Ren S, Li S, Kim TH, et al. Type V Collagen in Scar Tissue Regulates the Size of Scar after Heart Injury. Cell. 2020; 182: 545-62 e23.
